# Feasibility of Coacervate-Like Nanostructure for Instant Drug Nanoformulation

**DOI:** 10.1021/acsami.2c21586

**Published:** 2023-03-28

**Authors:** Geyunjian H. Zhu, Mohammad Azharuddin, Bapan Pramanik, Karin Roberg, Sujoy Kumar Biswas, Padraig D’arcy, Meng Lu, Apanpreet Kaur, Alexander Chen, Ashis Kumar Dhara, Alexandru Chivu, Yunhui Zhuang, Andrew Baker, Xiewen Liu, David Fairen-Jimenez, Bismoy Mazumder, Rongjun Chen, Clemens F. Kaminski, Gabriele S. Kaminski Schierle, Jorma Hinkula, Nigel K. H. Slater, Hirak K. Patra

**Affiliations:** † Department of Chemical Engineering and Biotechnology, University of Cambridge, Cambridge CB3 0AS, United Kingdom; ‡ Department of Biomedical and Clinical Sciences (BKV), Linkoping University, Linköping 58183, Sweden; § Department of Chemistry, Ben Gurion University of the Negev, Be’er Sheva 84105, Israel; ∥ Department of Otorhinolaryngology in Linköping, Anaesthetics, Operations and Specialty Surgery Center, Linköping University Hospital, Region Östergötland, Linköping 58185, Sweden; ⊥ AIMP Laboratories, C86 Baishnabghata, Patuli Township, Kolkata 700094, India; # Department of Chemical Engineering, Imperial College London, South Kensington Campus, London SW7 2AZ, United Kingdom; ¶ Department of Electrical Engineering, National Institute of Technology Durgapur, Durgapur 713209, West Bengal, India; ◆ Department of Surgical Biotechnology, Division of Surgery and Interventional Science, University College London, London NW3 2PF, United Kingdom

**Keywords:** nanomedicine, self-assembly, 3D tumor spheroids, coacervate-like nanostructure, instant nanoformulations

## Abstract

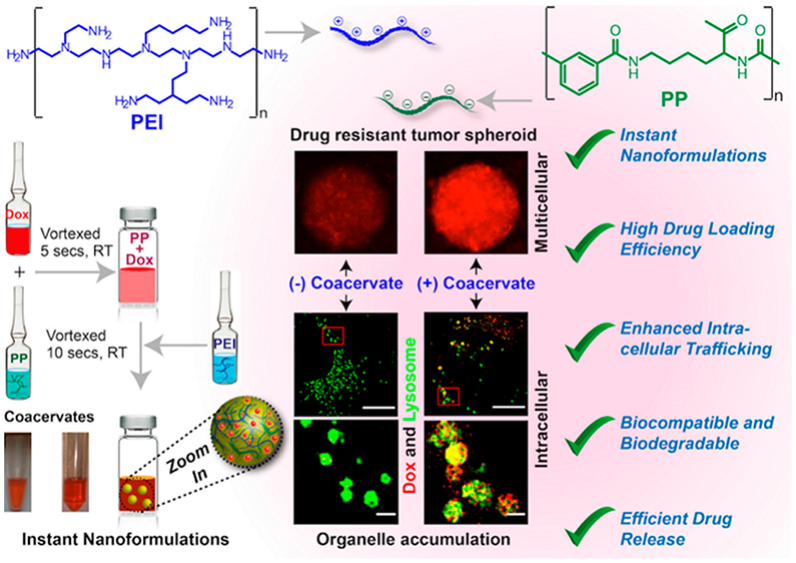

Despite the enormous advancements in nanomedicine research, a limited number of nanoformulations are available on the market, and few have been translated to clinics. An easily scalable, sustainable, and cost-effective manufacturing strategy and long-term stability for storage are crucial for successful translation. Here, we report a system and method to instantly formulate NF achieved with a nanoscale polyelectrolyte coacervate-like system, consisting of anionic pseudopeptide poly(l-lysine isophthalamide) derivatives, polyethylenimine, and doxorubicin (Dox) via simple “mix-and-go” addition of precursor solutions in seconds. The coacervate-like nanosystem shows enhanced intracellular delivery of Dox to patient-derived multidrug-resistant (MDR) cells in 3D tumor spheroids. The results demonstrate the feasibility of an instant drug formulation using a coacervate-like nanosystem. We envisage that this technique can be widely utilized in the nanomedicine field to bypass the special requirement of large-scale production and elongated shelf life of nanomaterials.

## Introduction

Nanosized materials can provide unique interactions with biological systems that can enable designing novel delivery systems,^1^ diagnostic strategies,^2^ and biosensors.^3^ However, large-scale manufacturing and storage requirements for nanoformulations (NF) impose severe difficulties in their translation, commercialization, and clinical application. Readily scalable nanoscale fabrication methods, such as laser ablation and lithography, suffer severe limitations due to the requirement of complex equipment, facilities and operation, as well as time and energy consumption, thereby impeding their range of applicability.^4^ Other, more cost-effective fabrication methods, such as wet chemical synthesis and nanomicelles, are routinely utilized to make colloidally stable nanoparticles in laboratory settings but can be difficult to scale up to the industrial level for biomedical applications.^5^ In addition to very high fabrication costs and difficult size control, lifetime and longevity are also a paramount concern. The long-term storage of nanoparticles can compromise their morphology, stability, and functionality.^6^

One innovative approach to overcoming the manufacturing and storage issues of nanomedicines is to develop instant NF (INF), which can be prepared at the bedside, immediately before administration, from precursors in a “mix-and-go” fashion. Inorganic nanoparticles are usually synthesized using a special apparatus or via wet chemical reactions with a bottom up approach in strict conditions, so they are not suitable for instant formulation of nanoparticles.^7,8^ Rather, polymeric nanoparticles can be fabricated spontaneously through the self-assembly process and therefore require minimum user intervention.^9−11^ In this regard, polyelectrolyte complexation has emerged as an attractive strategy due to its instantaneous nature, which occurs directly upon mixing of components. Depending on the fabrication condition, either liquid–solid phase separation (solid precipitate)^12^ or liquid–liquid phase separation (coacervate)^13^ in nano to submicron scale can be obtained. Coacervate has been recognized as an effective way to compartmentalize macromolecules in aqueous systems without the presence of membranes.^14^ It is also believed, according to the Oparin–Haldane hypothesis, that it could be an important mechanism to form protocells as a step in the origination of life.^15^ Coacervate systems have previously been utilized in hydrophobic drug dissolution,^16^ protein delivery,^17,18^ wound healing,^19,20^ angiogenesis enhancement,^21^ antibiotic delivery,^22^ and heart repair.^23^

**Scheme 1:**
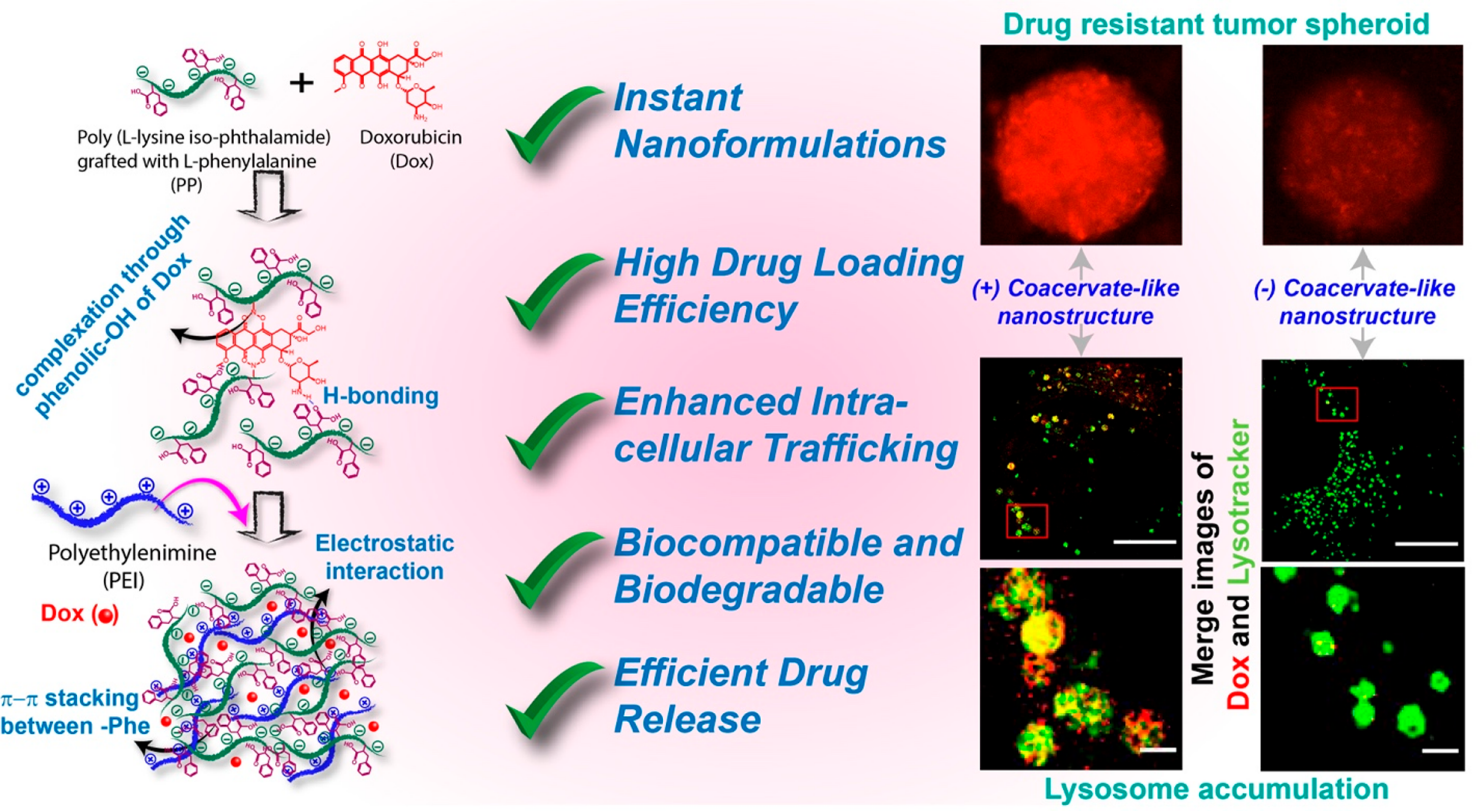
Instant Nanoformulation Strategies and Their Application in Cancer Treatments

Herein, we report the INF of a chemotherapeutic drug, doxorubicin (Dox), by employing an anionic pseudopeptide poly(l-lysine iso-phthalamide) grafted with l-phenylalanine (PP), a bioinspired polymer mimicking the amphiphilic structure and pH-responsive membrane-permeable and endosomolytic peptide as reported previously.^24^ The PP polymer has also been shown to facilitate the intracellular trafficking of payloads by enhanced endosomal escape.^25−30^ Due to the presence of the ionizable carboxylic acid groups, the PP polymer is a highly suitable candidate to form coacervate-like systems. In this proof-of-concept study, three types of PP polymers (PP25, PP50, and PP75) are optimized together with polyethylenimine (PEI) to form coacervate-like nano systems with Dox via a simple addition strategy (Scheme 1).

## Results and Discussion

**Figure 1:**
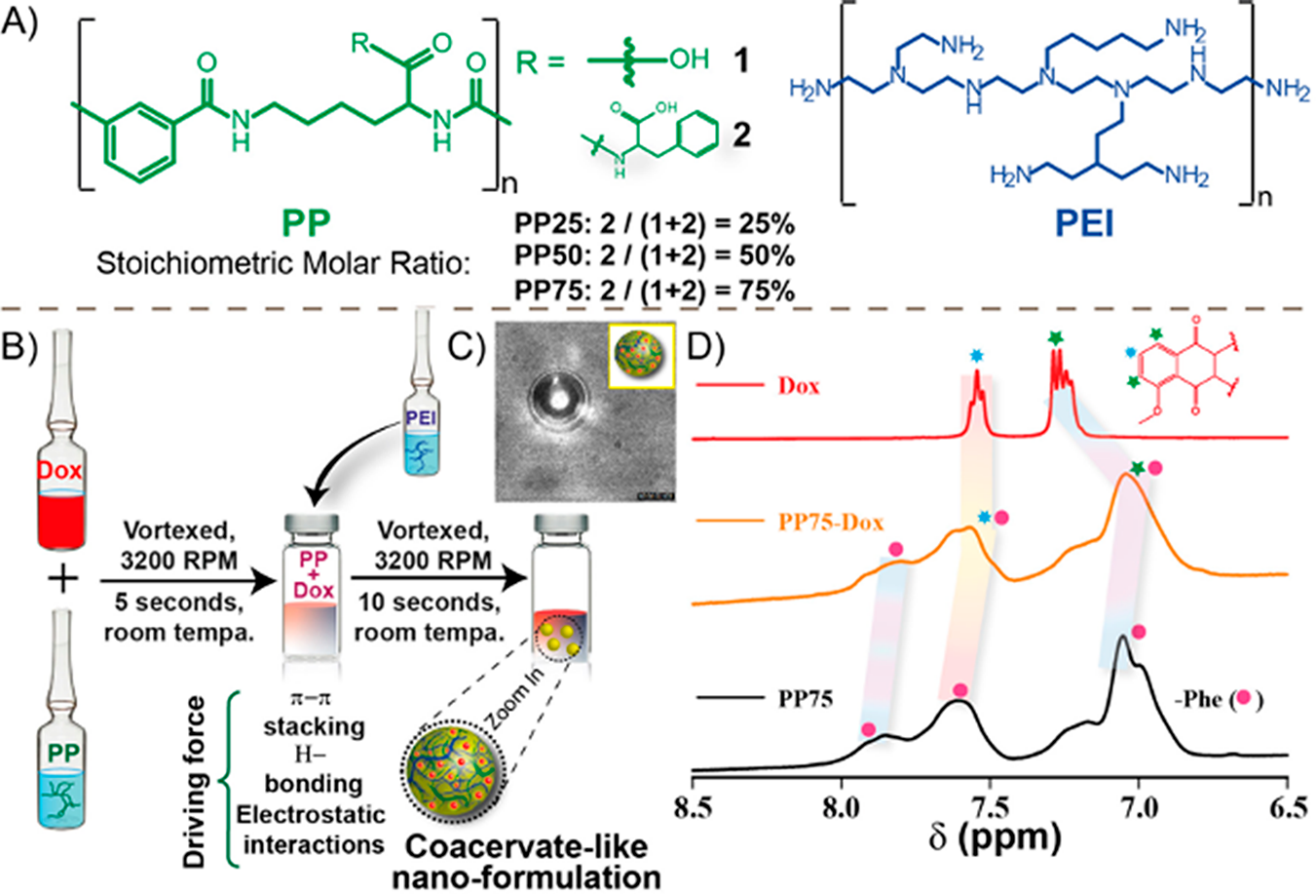
Fabrication process and assembly driving forces. (A) Chemical structures of PP polymers (PP50 and PP75) and PEI used in this study; (B) Pictorial presentation of coacervate-like nanoformulation preparation by simple mixing of anionic polymer (PP), cationic polymer (PEI) and chemotherapeutic drug, doxorubicin (Dox) in PBS buffer (pH 7.4); (C) Representative droplet like structure and (D) ^1^H NMR spectra of the of PP75, Dox and their mixture in D_2_O displaying upfield shifts as well as the broadening of aromatic protons due to stacking in aromatic regions.

PP25, PP50, and PP75 were used to create the coacervate-like nano system, where the numbers (25, 50, and 75) denote the stoichiometric substitution percentages of l-phenylalanine (-Phe) relative to pendant carboxylic acid (−COOH) groups along the backbone (Figure 1A). The precise degrees of substitution were 41.4 and 63.4 mol %, respectively, for PP50 and PP75, based on NMR spectra (Figure S1). The coacervation was achieved by the simple addition of PP, PEI, and Dox to PBS (Figure 1B). Electrostatic interaction between the cationic and anionic polymers, π–π interaction between the Phe and Dox, and H-bonding are plausible driving forces contributing to the coacervate-like system formation.^31^ To check the H-bonding and π–π-stacking, we investigated the ^1^H NMR spectral shift. In the presence of Dox, a prominent upfield shift of Dox as well as PP75 with broadening of -Phe protons was observed, indicating probable π–π stacking between phenyl rings of PP75 and Dox during the self-assembly process (Figure 1C).^32^ We were unable to find any −COOH signal because of the low solubility of PP75 as well as the intensification of −COOH in D_2_O. In the case of PP50, there was a lower upfield shift of -Phe protons, due to the difference in the hydrophobicity between the PP75 polymer and PP50 (Figure 1C and S2).^33,34^ The detailed interactions to form the coacervate-like system are clearly explained in the ESI (Figures S2 and S5).

**Figure 2:**
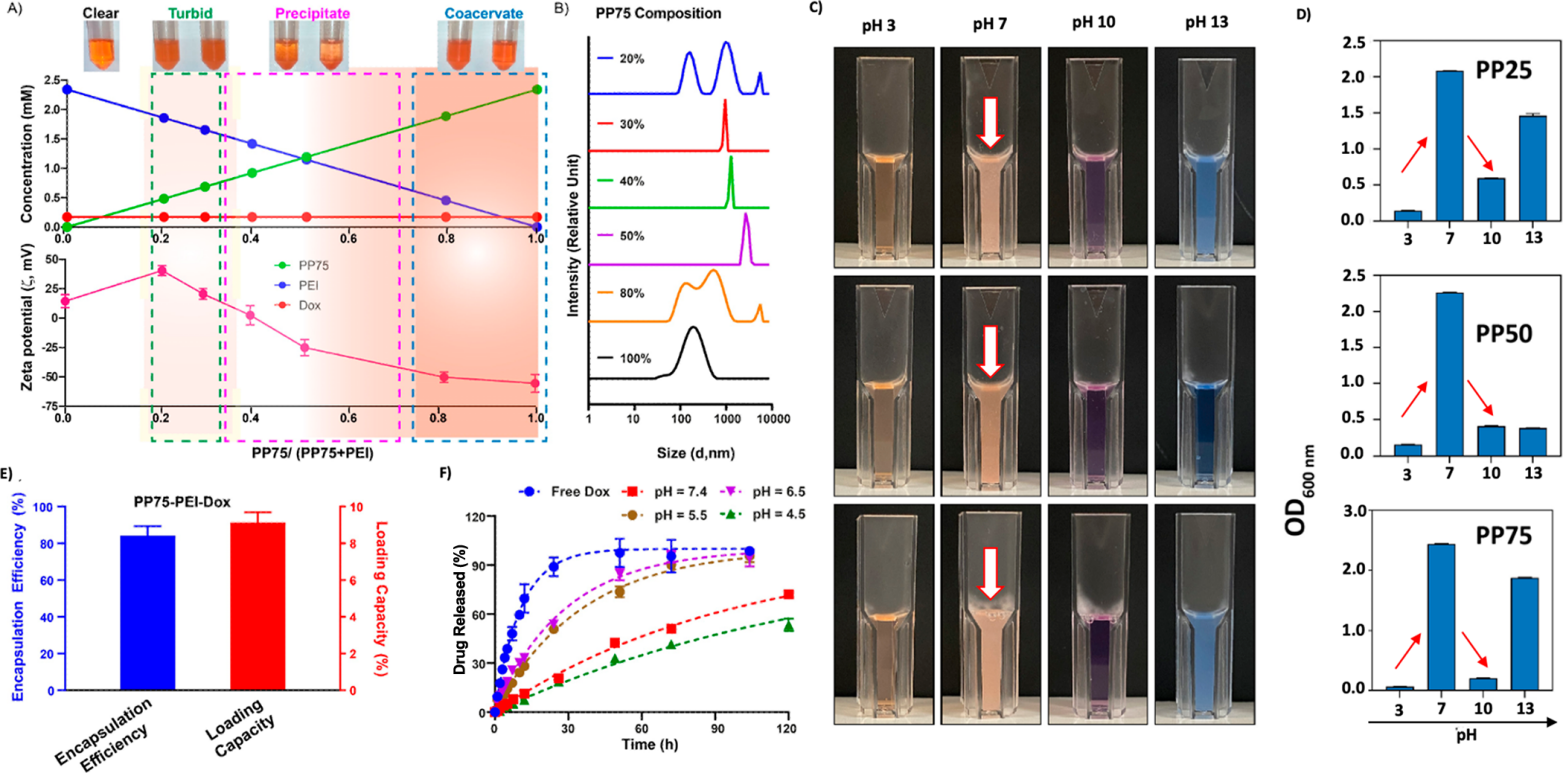
Optimization and characterization of the coacervate delivery system. (A) Photographs of different formulations made with various PP75:PEI ratios are shown in the top panel. Clear solution was obtained with only PEI + Dox. Precipitates were obtained with a mixing ratio close to 1. Unstable turbid mixtures resulting in a visible precipitate within an hour after blending were obtained with an overload of PEI, and stable turbid mixtures were obtained with an overload of PP75. Respective ζ-potential of complexes/coacervate-like nano system with different formulations are exhibited in the bottom panel. (B) Hydrodynamic diameters of complex coacervate-like nano system measured by DLS (PP75 composition is defined as [PP75]/([PP75] + [PEI])). (C) pH dependent optimal pH for different PPs (PP25, PP50 and PP75) coacervate-like nano system and high pH precipitation and drug instability (D). (E) Encapsulation efficiency and loading capacity of Dox in the PP75-PEI-Dox with PP75 composition at 0.8 (*N* = 9). (F) Drug release profile of Dox from the nanoformulation in PBS with different pH. The lines show the fitted release curve using exponential plateau fitting model with an asymptote set to 100%. Error bars represent standard deviations.

**Figure 3:**
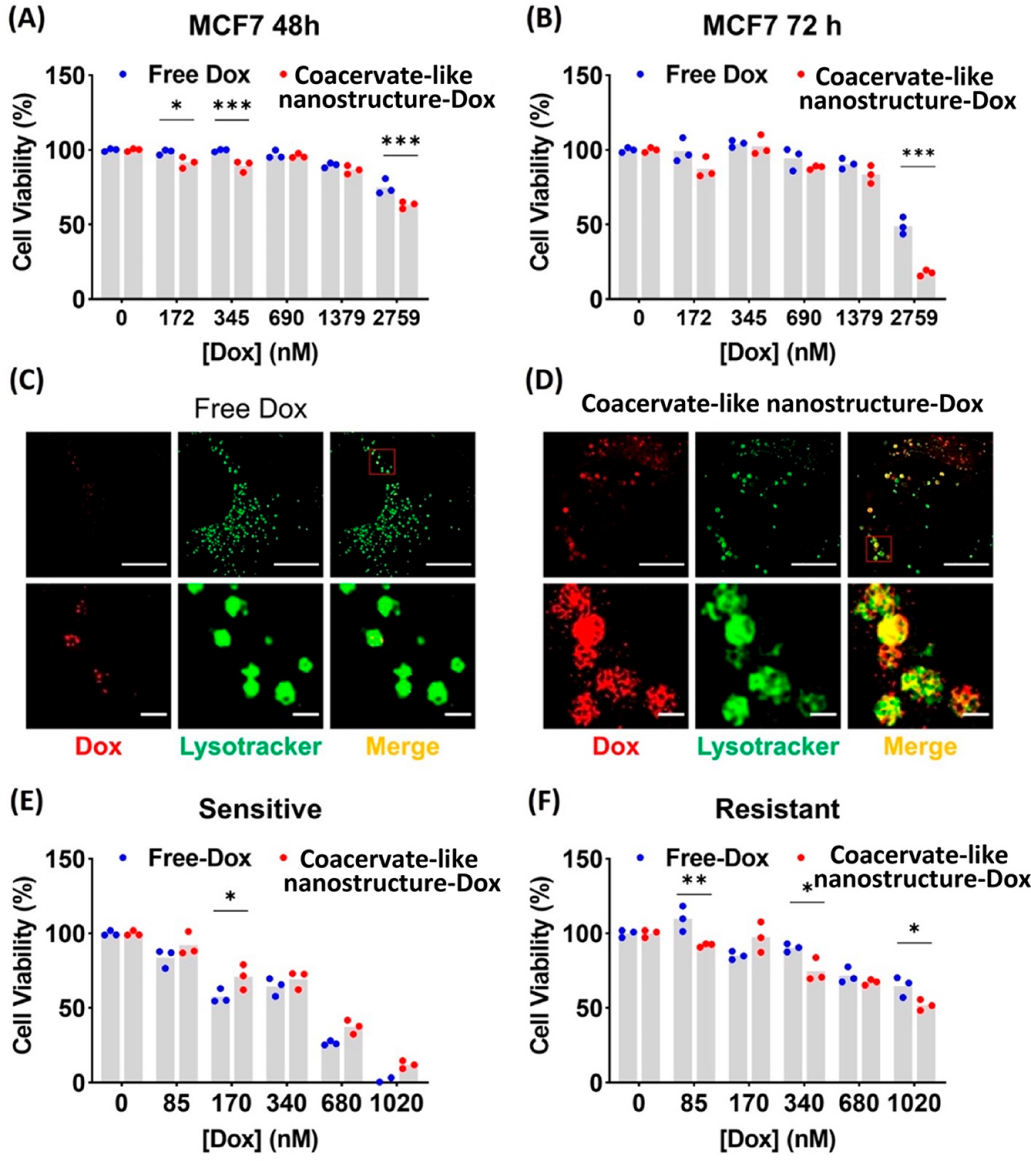
Delivery efficiency and endocytosis pathway of the coacervate-like INF system. Cytotoxic effects of free Dox and coacervate-like nanoformulation delivered Dox in MCF7 cell line after (A) 48 h and (B) 72 h of treatment as measured by MTS assay in 2D monolayer. SIM images of MCF7 treated with either (C) free Dox or (D) coacervate (red fluorescence) and incubated with Lysotracker (green fluorescence) for 5 h. Scale bars represent 10 μm (top) and 1 μm (bottom). Cytotoxic effects of free Dox and coacervate-like nanoformulation delivered Dox in (E) LK0917 (sensitive) and (F) LK1108 (resistant) 3D multicellular spheroids for 72 h of treatment (*n* = 3, *p ≤ 0.05, **p ≤ 0.01, ***p ≤ 0.001).

We have rationally developed the pseudo peptide like polymers PP (PP25, PP50, and PP75) with the indicated pendent ratios for a systematic study obtaining coacervate-like nano systems formulated with different stoichiometric ratios between and PEI (Figure 2A). The combined polymer concentration was held constant at 2.6 mM with respect to the repeating unit, while the Dox concentration was kept at 172 μM. The turbid (opaque) mixtures were obtained with appropriate composition of individual PP polymer and PEI (Figure 2A), where the composition of individual PP is defined as ([PP]/([PP]+[PEI]). However, mixtures with excess PEI resulted in a visible precipitate within 1 h of preparation and were not able to be redispersed upon vigorous agitation. The formulation strategy was tested at different pHs (Figure 2B) to study the physicochemical behavior. The visual inspection and quantitative turbidity experiment were performed, which indicated rich coacervate-like systems at pH 7 (Figure 2C). However, we have observed that at higher pH, the drug doxorubicin changes its physicochemical behavior (e.g., color changing to purple), and furthermore, the complex is precipitating at high pH like big chain polymers. Therefore, we showed that the optimality exists at pH 7. The zeta potentials (ζ) and nanoscale hydrodynamic diameters (H_D_) of each formulation with different stoichiometric composition ratios were measured by Dynamic Light Scattering (DLS) and quantitatively analyzed and tracked through Nanoparticle Tracking Analysis (NTA) (Figure 3A (bottom), B; Figures S4 and S5). The ζ-value of the complexes decreases with an increasing amount of PP75. The large negative values in the two formulations with highest PP75 compositions (0.8 and 1) provide high colloidal stability. Loading of Dox into the coacervate-like system was accomplished mainly through interactions between PP and Dox, therefore we preferred to incorporate higher content of PP75 in the system to maximize the loading efficiency. The addition of PEI is crucial to form the tertiary interaction to finalize the coacervate-like system and was found to achieve more efficient release of Dox than the PP75-Dox system. Second, the rationale is to include PEI in the nano system to enhance the intracellular trafficking of the payload due to PEI’s proton sponge effects.^35^ Therefore, the formulation with PP75 composition at 0.8 was selected for further investigation.

The hydrodynamic diameter of the nanoformulation with PP75 composition of 0.8 exhibited a two-peak distribution pattern, with one close to 100 nm and the second approaching 1 μm. A parallel optimization step was conducted with PP50 coacervate-like nanoformulation where similar two-peak distribution patterns were observed (Figure S6). This wide particle size distribution is reported for other coacervate-like systems, where small droplets can coalesce to form larger droplets over time, finally leading to bulk phase separation.^36−38^

The morphology of coacervate-like nanoformulation was then analyzed using scanning electron microscopy (SEM), transmission electron microscopy (TEM), and optical/fluorescence microscopy (Figure S7). The optical microscopic techniques (Figure S7) is not ideal for observing a nano scale object; however, it spotted the formation of droplet-like structures characteristic of coacervate with sizes ranging from 10 to 100 nm, consistent with the dynamic light scattering (DLS) measurements, indicating a coacervate-rich phase.^39^ We also attempted to visualize submicron spherical structures under the fluorescence microscopes, interestingly, at least confirming the successful encapsulation of Dox through their characteristic red fluorescent color. The successful encapsulation of Dox was further confirmed through a specialized ZetaView fluorescence filtered NTA imaging system with signals arising from the loaded coacervate-like systems (Figure S8). In an ideal scenario, Fluorescence Recovery after Photobleaching (FRAP) analysis of an individual coacervate droplet would have been an appropriate experiment to confirm our coacervate-like system to coacervate. Unfortunately, in our case, first, due to nanoscale size, it is impossible to see and follow an individual droplet through a fluorescent microscope (resolution limiting). Second, incorporating the fluorescent molecules at the PP backbone or pendant will change the entire physicochemical behavior of the PP. While we attempted electron microscopy, we can see that the individual nanosystems are not that helpful as the preprocessing of samples, high vacuum, and dried conditions make it impossible to check different phases. In terms of the encapsulation efficiency and loading capacity, the PP75-PEI is capable of encapsulating 84.2 ± 5.0% of the Dox added in the mixture, corresponding to 9.1 ± 0.5% loading capacity by weight (Figure 3C). The abilities of PP75 and PP50 to complex with Dox were compared without the presence of PEI (Figure S9). The PP75 with more l-phenylalanine substitution was capable of sequestering more Dox than PP50, indicating the participation of π–π interaction in the coacervation process. Due to the higher encapsulating efficiency and stability, the PP75 coacervate-like nanoformulation was subject to further investigation. The Dox release profiles from PP75-PEI-Dox nanoformulation were established in PBS with various adjusted pH conditions (Figure 3D). The coacervate-like system showed a significantly higher release rate under slightly acidic conditions (pH = 5.5 and 6.5) than at neutral pH, which corresponds to early late to late endosomes. Interestingly, the release rate was lowest below pH 4.5, even slower than at neutral pH, which might be caused by the low solubility of the PP75 at that pH.

The delivery efficiency of the PP-PEI-Dox coacervate-like nano system was evaluated. First, the range of nontoxic dose for the empty coacervate-like vehicles was established (Figure S10). The delivery of Dox by coacervate was then examined with MCF7 cells and two other breast cancer cell lines. Enhanced cytotoxicity was observed with the utilization of the coacervate delivery system at specific dosages (Figure 3A,B; Figure S11). Our team members already reported that the intracellular trafficking of PP polymers with covalently conjugated payloads are mainly delivered via the endolysosomal pathway, specifically through clathrin-mediated endocytosis.^25^ To confirm that the coacervate-like system undergoes a similar endocytic process, colocalization of Dox/coacervate-like nanoformulation with lysosomes/late endosomes were performed using structured illumination microscopy (SIM),^40^ which enables us to visualize the structure of lysosomes and their contents at around 100 nm resolution. The free Dox shows a low degree of colocalization with lysosomes (Figure 3C). The small amount of Dox residing in the lysosomes in the case of the free Dox group might be due to the sequestration of Dox in the organelles. Because of the basic and hydrophobic nature of the drug, Dox seems to accumulate in acidic organelles to some extent, such as lysosomes, where Dox can get protonated and lose membrane permeability.^41^ This mechanism is considered to cause drug resistance and has been argued to be one of the reasons for the inefficiency in the liposomal Dox formulation, Doxil.^42,43^ In comparison, Dox delivered by the developed nano system can be seen to closely colocalize with lysosomes. In addition, there was also substantial red fluorescence diffusing around the lysosomes in the coacervate-like group. With a longer treatment time, more dispersed red fluorescence can be observed around the lysosomes. This was likely the result of the Dox leaving the endo/lysosomes via PP-mediated lysosomal escape. Both free and coacervate delivered Dox ultimately accumulate in the nuclei (Figure S12).

The intracellular trafficking property of the PP polymer makes the coacervate-like nanoformulation system a suitable delivery vehicle against multi drug resistant (MDR) characteristics exhibited in drug resistant cancers. These cells overexpress drug efflux pump proteins, such as p-glycoproteins, which can pump out foreign substances including chemotherapeutic agents. One way to bypass the efflux pump is to utilize a delivery system that can enter the cells through a mechanism other than simple diffusion.^44^ Our previous reports demonstrated the limitation of 2D monolayer models in reflecting the level of resistance in MDR cells; therefore, we have selected the patient-derived pretreated postdiagnosed 3D multicellular tumor spheroid as our study model for investigating the efficacy of the coacervate-like nanoformulation in those MDR cancer cells.^45^ We have also shown that patient-derived head and neck cancer cell lines exhibiting different degrees of MDR work as an ideal study model to test and compare with the drug-sensitive (LK0917) and drug-resistant (LK1108) tumor spheroids.^45^

**Figure 4:**
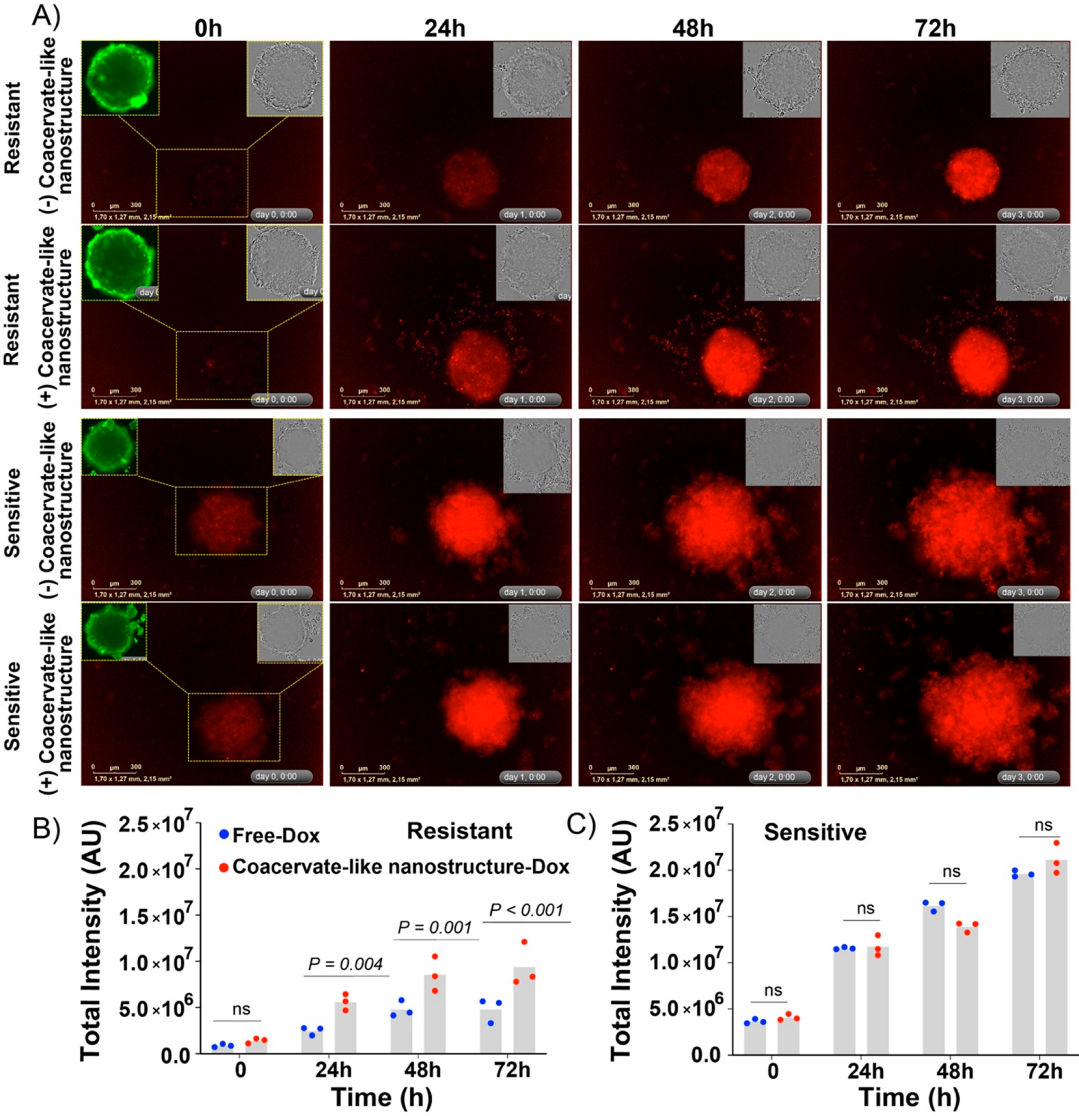
Real-time imaging of 3D tumor spheroids made with drug-sensitive (LK0917) and drug-resistant (LK1108) cancer cell lines over 72 h treatment. (A) Fluorescence images of resistant (top two rows) and sensitive (bottom two rows) spheroids treated with free Dox and coacervate-like nanoformulation at [Dox] = 800 nM at various time points. The insets show the calcein intake and bright field of the spheroids. (B,C) The corresponding fluorescence within the spheroids (*n* = 3).

Before the treatment, the 3D MDR model was validated with calcein probes (Figure S13). The coacervate-like nanoformulation showed similar cytotoxic effects as free Dox in sensitive spheroids and more effective cell-killing than free Dox in resistant spheroids, especially at 85, 340, and 1020 nM doses. To further confirm the delivery efficiency of the coacervate-like nanoformulation, a real-time fluorescence microscopic imaging study was performed with 3D cellular spheroids, and respective fluorescence intensities within the spheroids were analyzed (Figures 4 and S13–S14). The sensitive spheroid started to disintegrate after 48 h of treatment, while the resistant spheroids were able to stay relatively intact due to higher drug tolerance. The coacervate achieved a much higher accumulation and retention of Dox in the MDR spheroids from 24 to 72 h. In contrast, no noticeable differences in Dox uptake between free drug and the coacervate system were detected in drug sensitive spheroids, which agrees with cytotoxicity data. The enhanced delivery of Dox into 3D MDR tumor spheroids demonstrates the potential of the PP75-PEI coacervate-like instant nanoformulation as a system in MDR cancer therapy.

## Conclusion

In conclusion, we have demonstrated a system and method for the instant nanoformulation of a drug that can be enabled by a coacervation-like system. The PP-PEI-Dox system developed in this report exhibits a high encapsulation efficiency of Dox and can release it upon pH stimuli. The improved efficacy of Dox was observed against both drug-sensitive and drug-resistant patient-derived head and neck cancer cell lines in both 2D and 3D models. Real-time imaging of 3D tumor spheroids also suggests that the coacervate delivery system can facilitate the penetration and retention of the drug Dox into the tumor spheroid structure. Both the results validate great promise for the coacervate-based nano system as a tool to formulate a drug delivery system instantly which can fight against MDR cancer cells. With the careful selection of polymers and therapeutic agents, instant formulation of delivery systems with a wide range of functionalities and applications can be achieved in the future. With further in vivo validation, we envision that this method would become a platform technology for making bedside nanoformulations of a wide range of drugs.

## Experimental Section

### Raw Materials

Branched polyethylenimine (PEI) (*M*_w_ ∼ 25,000 *M*_n_ ∼ 10,000) was purchased from Sigma-Aldrich (Merk) and used without further purification. Doxorubicin Hydrochloride (Dox) was purchased from Tokyo Chemical Industry and Sigma-Aldrich and used as received. Dulbecco’s Modified Eagle Medium (DMEM), Dulbecco’s phosphate-buffered saline (DPBS), trypsin-EDTA, penicillin streptomycin (Pen Strep), and fetal bovine serum (FBS) were purchased from Thermo Fisher. CelTiter96 AQueous One Solution Cell Proliferation Assay was acquired from Promega. All solvents were procured from Sigma-Aldrich of Merck. To prepare the samples for the experiments, Milli-Q water with a conductivity of less than 2 μS cm^–1^ was used. ^1^H NMR spectra were recorded on a Bruker Ascend 500 MHz instrument (Bruker, Coventry, UK).

### Synthesis of PP Polymer

PP polymers were synthesized in-house according to a previously established procedure.^24,33^ Poly(l-Lysine Iso-phthalamide) (PLP) (Mw = 35,700, Mn = 17,900, polydispersity = 1.99) was grafted with different amounts of L- phenylalanine (Phe) to prepare the PP polymers. The numbers 50 and 75 represent the stoichiometric molar percentages of Phe relative to pendant carboxylic acid groups on the backbone of PLP. The actual degrees of grafting of PP50, and PP75 were determined from ^1^H NMR spectra, and it was found to be 41.4 and 63.4 mol %, respectively.

### Fabrication and Optimization of Coacervate-Like Nanoformulation

PP polymers and PEI were first dissolved in DPBS at a concentration of 5 mM with respect to the repeating unit. Dox was dissolved separately in DI-water at 1 mg/mL. To make the coacervate-like formulation, a predetermined volume of Dox stock solution was added to the PP polymer solution. The mixture was vortexed (Vortex-Genie 2) at 3200 rpm for at least 5 s. The PEI stock solution was added to the mixture, and the mixture was again vortexed for about 10 s. Similar procedures were used to make control samples of PP-Dox and PEI-Dox by replacing the PP and the PEI stock solution with PBS solution while holding the Dox to polymer ratio the same as in the coacervate samples. The coacervate-like nanoformulation can be used directly as prepared. However, to strictly compare the loaded and free Dox, the nanoformulation was dialyzed (Slide-A-Lyzer MINI Dialysis Devices) against DPBS overnight. The exact amount of Dox remaining in the system was calculated with quantification of Dox in the dialysis solution by measuring the fluorescence intensity with an emission signal at 590 nm and excitation at 470 nm (Tecan Spark Multimode Microplate Reader).

### Dynamic Light Scattering (DLS)/ζ-Potential Measurement

Hydrodynamic diameter and ζ-potential were measured at 25 °C with a Zetasizer Nano ZS (Malvern PANalytical Products, UK) with at least 90 scans for each sample. For coacervate-like samples, measurements were taken directly with the emulsion, and for samples with visible precipitate, measurements were taken on the supernatant solution.

### Nanoparticle Tracking Analysis (NTA)

NTA analysis was performed using PMX 220 ZetaView TWIN Laser equipment by ParticleMetrix GmbH and its corresponding software. The 520 nm excitation laser was set 90° from the CCD detector. A volume of 2 mL of each sample (neat for fluorescence filter signals, 200-fold diluted in deionized water for pure scattering signal) was injected into the quartz cell, and video acquisitions were collected of scattering signals at 11 different positions throughout the cell, with two cycles for each position. The instrument preacquisition parameters were initially optimized by the software and finally set to a temperature of 22 °C, sensitivity of 65, a frame rate of 30 frames per second, a shutter speed of 300, and a laser pulse duration equal to that of shutter duration. For fluorescence signal acquisition, a 540 nm filter was placed between the cell and the CCD detector.

### Transmission Electron Microscopy (TEM) Imaging

The TEM images were captured with a FEI TECNAI F20 instrument with an acceleration voltage of 200 kV. Samples were prepared by drop-casting the nanoformulation onto a 300 mesh Cu grid grit followed by air-drying overnight.

### Scanning Electron Microscopy (SEM) Imaging

SEM images were taken on a Nova nanoSEM instrument at 10 kV and a working distance of 6.3 mm. The samples were coated with platinum.

### Evaluation of Loading Capacity and Encapsulation Efficiency

Samples were centrifuged at 14,000*g* for 30 min before the Dox content in the supernatant was measured by fluorescence intensity with emission signal at 590 nm and excitation at 470 nm (Tecan Spark Multimode Microplate Reader). Loading capacity and encapsulation efficiency are defined as follows:
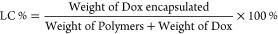
1
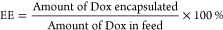
2

### Investigation on pH-Dependent Release Profile

As-prepared coacervate systems (0.5 mL) were pipetted into the Slide-A-Lyzer MINI Dialysis Devices against DPBS (14 mL) with adjusted pH. The dialysis devices were placed on a shaker for the entirety of the release experiment. At different time points, 1 mL of dialysate was taken from the device and replaced with 1 mL of DPBS with corresponding pH. The released Dox content in the dialysate was measured by fluorescence intensity.

### Stability Assessment of Coacervate-Like Nano Systems in Aqueous and BSA Solutions

Coacervate-like nano systems made with different compositions were compared for their relative colloidal stability in both DPBS and in the presence of Bovine Serum Albumin (BSA). Freshly made nanoformulations were diluted by 2 times with DPBS or BSA-containing DPBS and allowed to stand for 24 h. The absorbance of the samples immediately after the fabrication and after 24 h were measured. In the case of samples with precipitate, measurements were taken with supernatant. The relative absorbance was taken as a metric of colloidal stability.

### MTS Assay with MCF7, MDA-MD-231, and T47D Cell Lines

All cells were cultured in DMEM containing 10% FBS, 50 IU/mL penicillin, and 50 g/mL streptomycin and maintained at 37 °C in a humidified 5% CO2 incubator. The cells were seeded in 96-well plates at 10,000/well for 24 h and then treated with 100 μL of culture medium containing PP75-PEI-Dox, PP75-Dox, PEI-Dox, and free Dox at various Dox treating concentrations. After the respective treating time, the media was discarded. Each well was washed with 100 μL of DPBS followed by the addition of 120 μL of MTS solution (100 μL supplemented culture media + 20 μL CelTiter 96 Aqueous One Solution) and incubated at 37 °C, 5% CO_2_ for 1–4 h. The absorbance of each well was measured at 490 nm (650 nm as the reference wavelength) using a Tecan Spark Multimode Microplate Reader.

### Confocal Fluorescent Microscopy with MCF7 Treated with Coacervate

Measurements were carried out by using a Leica TCS SP5 confocal microscope. MCF7 cells were cultured on coverslips sitting at the bottom of the wells in 24-well plates. Cells were seeded at 5 × 10^4^ cells/well for 24 h and then treated with 0.5 mL culture media containing PP75-Dox, PP50-Dox, or free Dox at Dox concentration of either 0.8 or 1.6 μg/mL. After 12 h of treatment, the cells were fixed with 4% paraformaldehyde (Sigma-Aldrich) for 10 min. The coverslips were then mounted to microscope glass slides using ProLong Gold Antifade Mountant with DAPI (P36935, Thermo Fisher Scientific).

### Structured Illumination Microscopy (SIM) Study of Endocytosis of Coacervate-Like Nano System

SIM imaging was performed using a customer three-color system built around an Olympus IX71 microscope stage, as previously described.^40,46^ Laser wavelengths of 488 nm (iBEAMSMART- 488, Toptica), 561 nm (OBIS 561, Coherent), and 640 nm (MLD 640, Cobolt) were used to excite the fluorescence in the samples. A 60 Å∼/1.2 numerical aperture (NA) water immersion lens (UPLSAPO 60XW, Olympus) focused the structured illumination pattern onto the sample. This lens captured the samples’ fluorescent emission light before being imaged onto an sCMOS camera (C11440, Hamamatsu). Raw images were acquired with HCImage software (Hamamatsu). MCF7 cells were treated with PP75 coacervate-like nano system and free Dox (both with 6.4 μg/mL Dox concentration) for various time periods and then stained with LysotrackerTM according to the protocol provided by Thermofisher before they were imaged by SIM. Reconstruction of the SIM images with LAG SIM Resolution-enhanced images were reconstructed from the raw SIM data with LAG SIM, a custom plugin for Fiji/ImageJ available in the Fiji Updater. LAG SIM provides an interface to the Java functions provided by fair SIM.^47^

### MTS Assay with Patient-Derived Head and Neck Cancer Cell Lines

The cell lines sensitive and resistant used for this study were established from two different head and neck squamous cancer cell (HNSCC) patients as described previously.^45,48,49^ For the generation of tumor spheroids sized 300–500 μm, 200 μL of sensitive and resistant, single-cell suspensions were seeded in ULA plates (Corning Life Sciences) at varying cell densities in the range of 0.25–0.75 × 10^5^ cells/mL. The plates were incubated at a humidified 5% CO_2_ atmosphere at 37 °C (48–72 h) for formation of 3D tumor spheroids. Monolayers of sensitive and resistant cell lines were seeded in 96-well flat-bottom plates at a cell density of 8000 cells/well in 200 μL of complete medium at 37 °C and 5% CO_2_ atmosphere for 24 h before treatment. After 24 h, the culture medium was carefully aspirated, and monolayer cultures were treated with a coacervate-like nano system, PEI-Dox mixture or free Dox at 42.5 to 1020 nM Dox concentration. Cells were treated for 72 h. Sensitive and resistant spheroids were also treated with the same concentration used for the monolayer cell cultures, by replacing 50% of the culture medium with freshly prepared drug-supplemented medium,^50^ followed by incubation at 37 °C and 5% CO_2_ atmosphere for 72 h. Cell cytotoxicity in the drug-treated monolayer cell cultures was assessed using the CellTiter96 AQueous One Solution Cell Proliferation Assay (Promega). Briefly, at the end of 72 h, the drug supplemented medium was replaced with 317 μg/mL MTS reagent-supplemented medium. For a total volume of 200 μL, 40 μL of the MTS reagent was added into each well, and the plates were incubated at 37 °C and 5% CO_2_ atmosphere for 3 h. At the end of the incubation period, absorbances at 490 and 650 nm were recorded using a microplate reader (VersaMax, Molecular Devices). All experiments were performed in triplicates.

### Real-Time Live Cell Imaging of Dox Uptake in 3D Spheroids

The real-time calcein uptake and intracellular calcein accumulation in 3D sensitive and resistant tumor spheroids using cells from pretreated post diagnosed head and neck cancer cells.^45^ After spheroid formation, the keratinocyte serum-free growth medium (KSFM, Gibco, Thermo Fisher Scientific) was carefully decanted without disturbing the spheroids. They were then incubated in serum-free KSFM medium containing nonfluorescent calcein acetoxymethyl ester (calcein-AM, 1 mM in Dimethyl sulfoxide (DMSO), Sigma AB) at a final concentration of 1 μM and verapamil (MDR1 inhibitor, 20 μM), for 72 h at 37 °C and 5% CO_2_ atmosphere. During the 72 h incubation period, phase contrast and green fluorescence (Calcein Ex/Em = 495/515 nm) images of the spheroids were acquired every 30 min using time-lapse fluorescent microscopy. A 10× objective was used for image acquisition (Incucyte Zoom, Sartorius AG). The spheroids were also treated with free Dox and coacervate Dox ([Dox] = 50–800 nM), followed by live-cell imaging for red fluorescence as described above. Experiments were performed in at least triplicate for each individual study.

### Statistical Analysis

All of the experiments were conducted in triplicates. Statistical analysis was performed using the ANOVA in GraphPad Prism 8.0 (GraphPad Software, San Diego, USA), followed by Bonferroni *t* test for comparison with the untreated/control group. Statistical significance was determined at a *p ≤ 0.05, **p ≤ 0.01, and***P ≤ 0.001.

## Supporting Information Available

Investigation on the driving force of coacervate-like assembly (Figures S1–S3), nanoparticle tracking analysis (Figures S4–S5), optimization of mixing ratio for PP coacervate-like system (Figure S6), morphological characterization of coacervate system (Figure S7), fluorescence scattering signals from Dox-loaded complexes (Figure S8), comparison of loading of Dox between PP50 and PP75 (Figure S9), cytotoxic effects of Dox delivered by the coacervate-like nano system (Figures S10–S11), structured illumination and confocal microscopic studies (Figure S9), investigation of delivery effects of the nanoformulation in 3D multicellular spheroids (Figures S13–S15) (PDF)

## Dedication

We would like to dedicate this publication to all the frontline workers who continue to work relentlessly during the COVID-19 pandemic
