# Supplementary material for: Feasibility of Coacervate-Like Nanostructure for Instant Drug Nanoformulation
Source: ACS Appl Mater Interfaces. 2023 Mar 28;15(14):17485–94. doi: 10.1021/acsami.2c21586 (PMC10103128; doi:10.1021/acsami.2c21586)
Supplement: Supplementary file 1 — am2c21586_si_001.pdf [file am2c21586_si_001.pdf]

Supporting Information

Feasibility of coacervate-like nanostructure for instant drug nanoformulation

Geyunjian H. Zhu[‡,1], Mohammad Azharuddin[‡†,2], Bapan Pramanik[3], Karin Roberg[2,4], Sujoy Kumar Biswas[5], Padraig D'arcy, Meng Lu[1], Apanpreet Kaur[6], Alexander Chen[1], Ashis Kumar Dhara[7], Alexandru Chivu[8], Yunhui Zhuang[1], Andrew Baker[1], Xiewen Liu[1], David Fairen-Jimenez[1], Bismoy Mazumder[1], Rongjun Chen[6], Clemens F. Kaminski[1], Gabriele S. Kaminski Schierle [1], Jorma Hinkula[2], Nigel K. H. Slater[1], and Hirak K. Patra[,8]*

[1]Department of Chemical Engineering and Biotechnology, University of Cambridge, Philippa Fawcett Dr, Cambridge CB3 0AS, United Kingdom

[2]Department of Biomedical and Clinical Sciences (BKV), Linkoping University, Linköping 58183, Sweden

[3]Department of Chemistry, Ben Gurion University of the Negev, Be'er Sheva 84105, Israel

[4]Department of Otorhinolaryngology in Linköping, Anaesthetics, Operations and Specialty Surgery Center, Linköping University Hospital, Region Östergötland, Linköping 58185, Sweden

[5]AIMP Labs, C86 Baishnabghata Patuli Township, Kolkata 700094, India

[6]Department of Chemical Engineering, Imperial College London, South Kensington Campus, London SW7 2AZ, United Kingdom

[7]Department of Electrical Engineering, National Institute of Technology Durgapur, Durgapur 713209, West Bengal, India

[8]Department of Surgical Biotechnology, Division of Surgery and Interventional Science, University College London, London NW3 2PF, United Kingdom

Corresponding Author

*Hirak K. Patra

Email: hirak.patra@ucl.ac.uk

Tel: +44 20 3108 5658

Summary of content

Figure No.	Figure caption	Page No.
**Figure S1**	^{1}H NMR spectra of PP50 and PP75 in D_2O.	S-6
**Figure S2**	The aromatic region of ^{1}H NMR spectra of PP50, Dox and their mixture validated the role of phenyl group present in PP in the coacervate-like system in D_2O.	S-7
**Figure S3**	(A) Photographs of micro centrifuge tubes containing Dox solutions in DMSO with different amount of PP50 showing the color change at room temperature; (B) Partial ^{1}H NMR spectra of PP50, Dox and their mixture in DMSO-d_6; (C) UV-Vis spectra of Dox in the presence of different amounts of PP50. Inset of (C): Variation of absorption ratio (A_{608} nm/A_{482} nm) as a function of PP50 equivalence.	S-8
**Figure S4**	NTA hydrodynamic diameters for complexes formed in the presence of doxorubicin between different ratios of PP and PEI polyelectrolytes.	S-9
**Figure S5**	Nanoparticle scattering signals recorded on a ZetaView™ Twin nanoparticle tracking analyser for complexes of PP and PEI in the presence of Dox. Text legend: top row – PP polymer type; the numbers after PP denote % molar conjugation of the PP repeating unit with phenylalanine (Phe); side row – relative molar ratio of the PP polymer to the total polyelectrolyte concentration in the coacervate-like system mixture.	S-10
**Figure S6**	(A) Photographs of different formulas made with various PP50/PEI ratios are listed in the top panel. Clear solution was obtained with only PEI + Dox, precipitation was obtained with PP50 composition from 0.2 to 0.67, and turbid mixtures were obtained with PP50 composition greater than 0.75. Respective ζ value of complexes/coacervates-like structures with different formulations are exhibited in the bottom panel. (B) Hydrodynamic diameters of complexes/coacervates-like structures measured by DLS (PP50 composition is defined as [PP50] / ([PP50] + [PEI])).	S-11
**Figure S7**	(A) SEM of coacervate-like system made with PP75 to PEI ratio at 4:1 and Dox concentration at 0.1mg/mL B) TEM image of coacervate-like system made with PP50 to PEI ratio at 4 to 1 and Dox concentration at 0.1mg/mL (C) Bright field and fluorescence field (Dox) images of coacervate-like system made with PP50 to PEI ratio at 4 to 1 and Dox concentration at 0.1mg/mL.	S-12
**Figure S8**	(A) Encapsulation efficiency and (B) loading capacity of coacervates-like system/complexes made with various ratios of PP75+Dox and PP50+Dox without PEI. Stability of coacervate-like system made with various ratios of PP75+Dox and PP50+Dox in (C) PBS and (D)10 mg/mL BSA. Stability was evaluated by recording changes in UV-VIS absorbance of the supernatant at 480 nm after allowing samples to stand for 24 hr. Encapsulation efficiency of Dox in PP75-PEI-Dox coacervate-like system fabricated with (E) various mixing ratio and (F) different mixing components.	S-13
**Figure S9**	(a) SEM of coacervate-like system made with PP75 to PEI ratio at 4:1 and Dox concentration at 0.1mg/mL (b) TEM image of coacervate-like system made	S-14

	with PP50 to PEI ratio at 4 to 1 and Dox concentration at 0.1mg/mL (c) Bright field and fluorescence field (Dox) images of coacervate-like system made with PP50 to PEI ratio at 4 to 1 and Dox concentration at 0.1mg/mL.	
**Figure S10**	Cytotoxic effects of delivering agents PP75-PEI, PP75, and PEI measured with MTS assay against (A) MCF7 and (B) MDA231 (n = 3).	S-16
**Figure S11**	Dose-response curves measured by MTS assay with 2D monolayer of MCF7(A) & (B), MDA231(C) & (D), and T47D E) & (F) breast cancer cell lines treated with PP75-PEI-Dox, PP75-Dox, PEI-Dox, and free, respectively Dox at 48 and 72 hours. The IC_{50} values are summarized in the tabular form (G).	S-18
**Figure S12**	Structured illumination microscopy (SIM) of MCF7 treated with either free doxorubicin or coacervate-like system (red fluorescence) and incubated with Lysotracker Green (green fluorescence) at 1, 2, and 5 hours, showing the subcellular distribution of free Dox and coacervate-like system. Scale bar represents 10µm. (B) Localization and uptake of coacervate-like system (fabricated at 80% [PP]/([PP]+[PEI])) delivered Dox by MCF breast cancer cells.	S-19
**Figure S13**	Validation of 3D tumor Spheroid from pre-treated patient's cancer cell. (A) The corresponding bright field microscopic images of spheroids. (B) Confocal images of 3D tumor spheroids made with drug-sensitive and drug-resistant cancer cells treated with calcein at 0 and 120 minutes. Raw total green fluorescence intensity in the field of view as acquired by the microscope of control groups (-/+ verapamil inhibitor) measured over 72 hours of calcein-AM treatment in (C) sensitive and (D) resistant spheroids during real-time imaging of 3D spheroids.	S-20
**Figure S14**	Cytotoxic effects of free Dox and coacervate-like-Dox system in (A) sensitive (LK0917) and (B) resistant (LK1108) cell lines in 2D monolayer (***$p \leq 0.001$). Raw total red fluorescence intensity in the field of view as acquired by the microscope in (C) sensitive and (D) resistant spheroids treated with free Dox and nanocoacervate-like-Dox system over 72-hour treatment during real-time imaging of 3D spheroids.	S-22
**Figure S15**	Drug penetration: (A) Sensitive and resistant tumour spheroids were treated with Calcein AM (+/-) Verapamil (MDR1 efflux pump inhibitor). Real-time live-cell fluorescence imaging of the spheroids was obtained over a period of 72 hours. Green fluorescence intensity corresponding to calcein uptake is shown here as obtained from the Incucyte Zoom. Each panel represents a spheroid. (B) Live-cell real -time imaging of the spheroids treated with the drug (+/-) coacervate-like structures is shown here for sensitive and resistant spheroids.	S-23

Investigation on the driving force of coacervate-like assembly

NMR spectra of PP50 and PP75 in D_2O

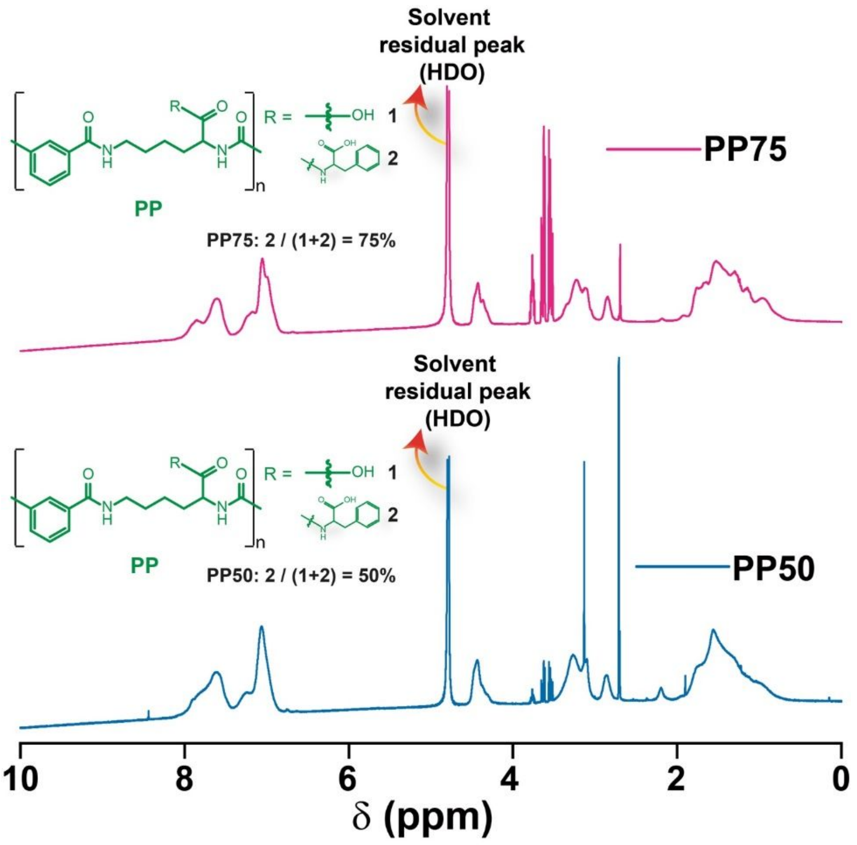

Figure S1. ^1H NMR spectra of PP50 and PP75 in D_2O.

^{1}H NMR spectra of aromatic region

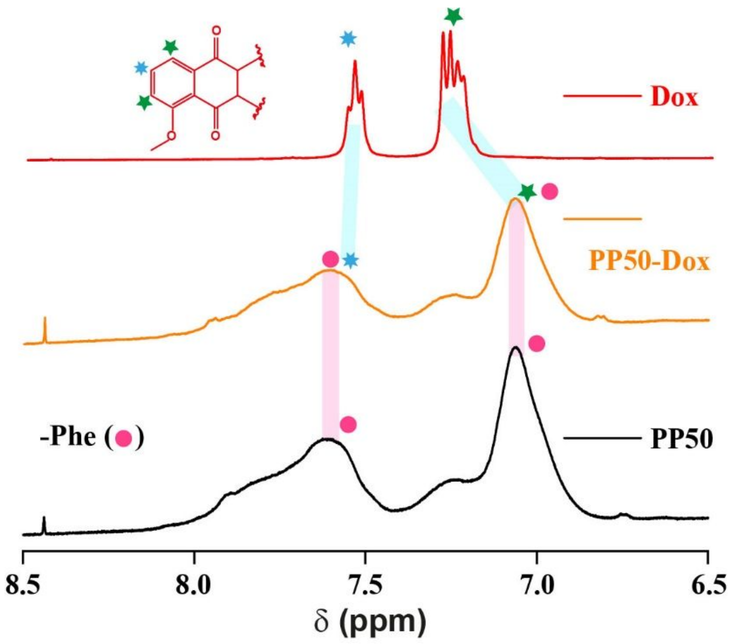

Figure S2. The aromatic region of ^{1}H NMR spectra of PP50, Dox and their mixture validated the role of phenyl group present in PP in the coacervate-like system in D_2O.

Because of the low solubility as well as -COOH peak intensification of PP in D_2O, NMR was measured in deuterated DMSO solvent (DMSO-d_6) to check the role of -COOH group present in PP as well as in Dox during coacervate-like system formation. Unexpectedly, -COOH signal cannot be observed in case of PP50, but in the presence of PP50, the -OH signals of Dox diminished and thus, confirmed the participation of -OH in the assembly process (**Figure S3b**). The substitution ratios were calculated from the molar ratio between the phenyl proton on the backbone at chemical shift around 7.5 ppm and the 5 phenyl protons on the substituted phenylalanine at chemical shift around 7.1 ppm based on the spectra obtained in DMSO-d6. The generation of deep-brown color from colorless PP50 solution also indicated a possible complex formation between PP50 and Dox (**Figure S3A**). The continuous decrease of absorbance at 482 nm with the concomitant enhancement of a new peak at a higher wavelength at 602 nm in UV-Vis

spectroscopy confirmed the active participation of -OH group of Dox in the coacervate-like system (**Figure S3C**).[1]

Formation of coacervate-like structures

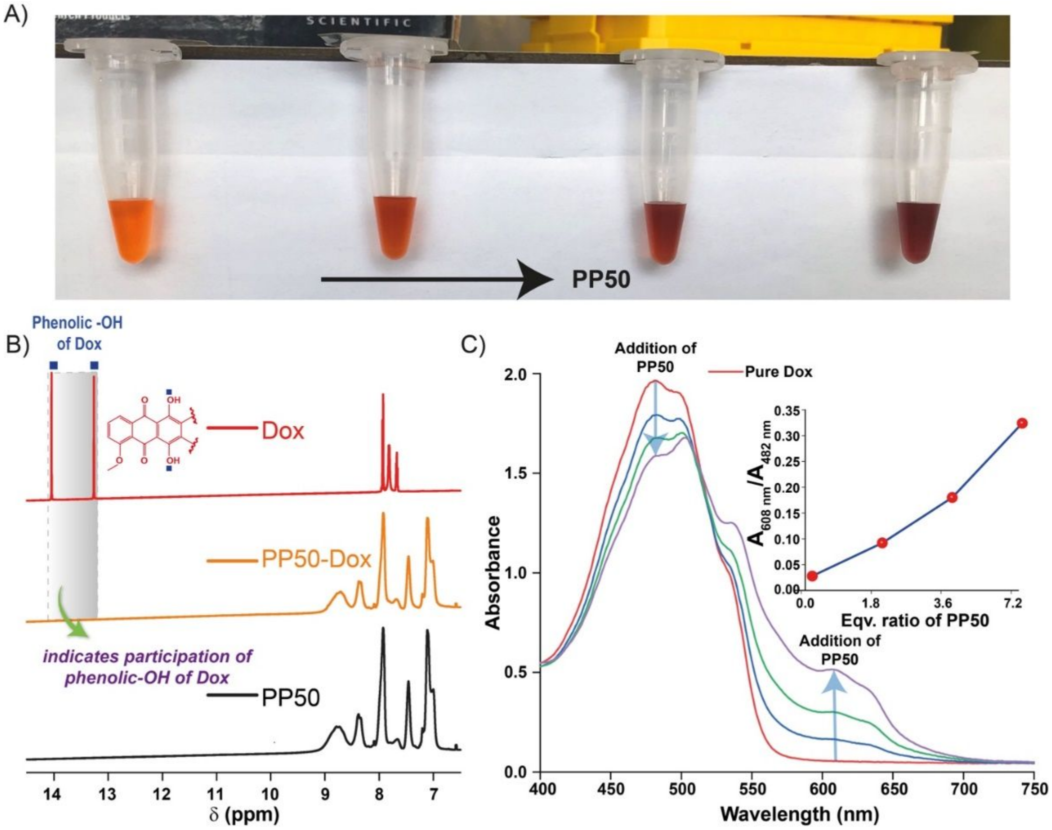

Figure S3. (A) Photographs of micro centrifuge tubes containing Dox solutions in DMSO with different amount of PP50 showing the color change at room temperature; (B) Partial 1H NMR spectra of PP50, Dox and their mixture in DMSO-d6; (C) UV-Vis spectra of Dox in the presence of different amounts of PP50. Inset of (C): Variation of absorption ratio (A608 nm/A482 nm) as a function of PP50 equivalence.

Nanoparticle tracking analysis (NTA)

Comparison of coacervate-like system hydrodynamic diameters

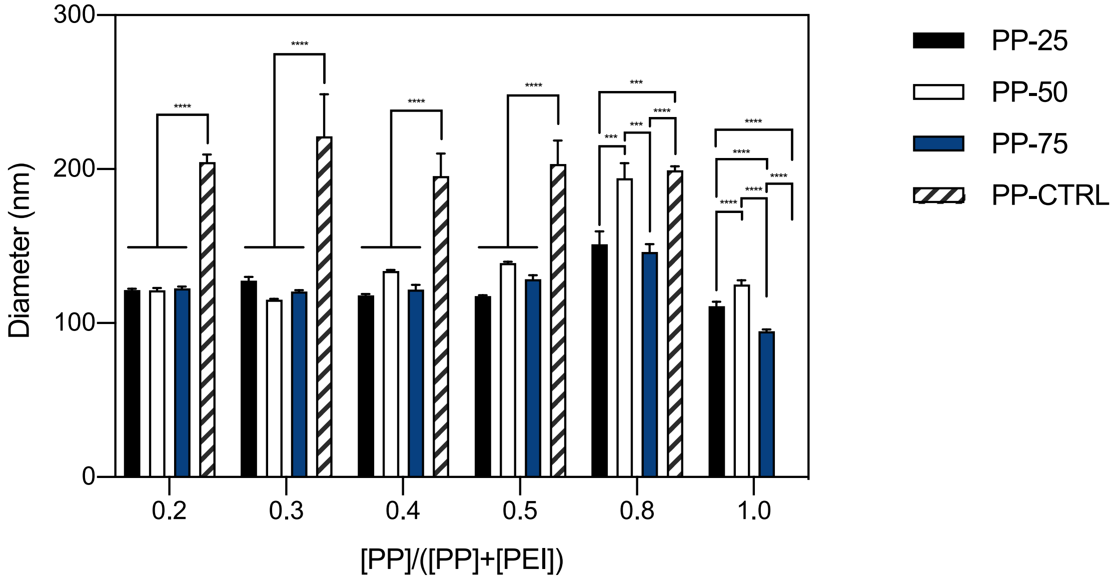

Figure S4. NTA hydrodynamic diameters for complexes formed in the presence of doxorubicin between different ratios of PP and PEI polyelectrolytes. In some conditions, the PP backbone was modified by the addition of phenylalanine (Phe) pendant groups at different molar ratios of Phe to the PP repeating unit (denoted in % by the numbers after the hyphen for the PP designation). The data was plotted using GraphPad Prism v9 and compared using ANOVA statistical analysis, considered significant for $p < 0.05$. The levels of significance are denoted as *** $p < 0.001$, **** $p < 0.0001$

The data was acquired by nanoparticle tracking analysis (NTA) and showed a marked difference in size ($p < 0.0001$) between the complexes formed by PEI and PP-Control (without Phe pendants) when compared to those formed by PEI and PP with Phe pendants (for all Phe contents, and for all PP:PEI ratios studied). For higher ratios of PP and PEI (e.g., 0.8), the effect of the Phe on coacervate-like structure size became more pronounced and there were also differences between the modified polymer coacervates-like system as well ($p < 0.001$). Unlike the Phe-conjugated PP, the control PP unable to form detectable particles without the presence of PEI (i.e., ratio 1 in **Figure S13**)

Scattering signals detected by the nanoparticle tracking analyser

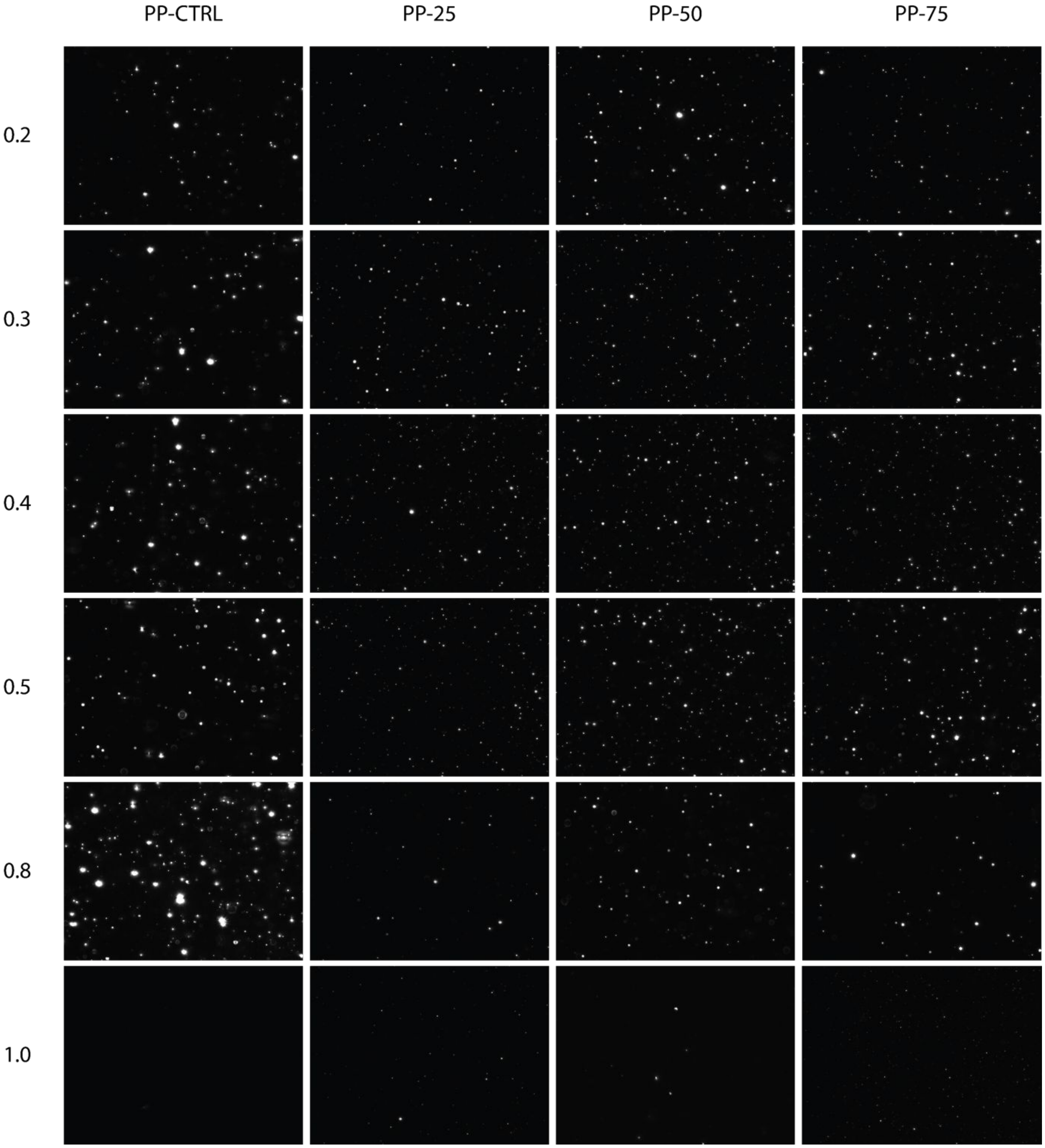

Figure S5. Nanoparticle scattering signals recorded on a ZetaView™ Twin nanoparticle tracking analyser for complexes of PP and PEI in the presence of Dox. Text legend: top row – PP polymer type; the numbers after PP denote % molar conjugation of the PP repeating unit with phenylalanine (Phe) side row – relative molar ratio of the PP polymer to the total polyelectrolyte concentration in the coacervate-like mixture.

The complexes were prepared as described text and further diluted 200-fold in deionised water 2 minutes after preparation and immediately injected into the equipment's quartz cell for signal

acquisition. Scattering signals were detectable for all PP polymers in the presence of PEI (i.e., ratios 0.2 – 0.8). Signals were also detected for Dox mixed with Phe-conjugated PP without any PEI (i.e., ratio 1.0) indicating colloidal self-assembly, but this signal was absent for the control/unmodified PP suggesting it was unable to interact with Dox similarly to Phe-modified analogues.

Optimization of mixing ratio for PP50 coacervate-like structures system

Coacervate-like system optimisation

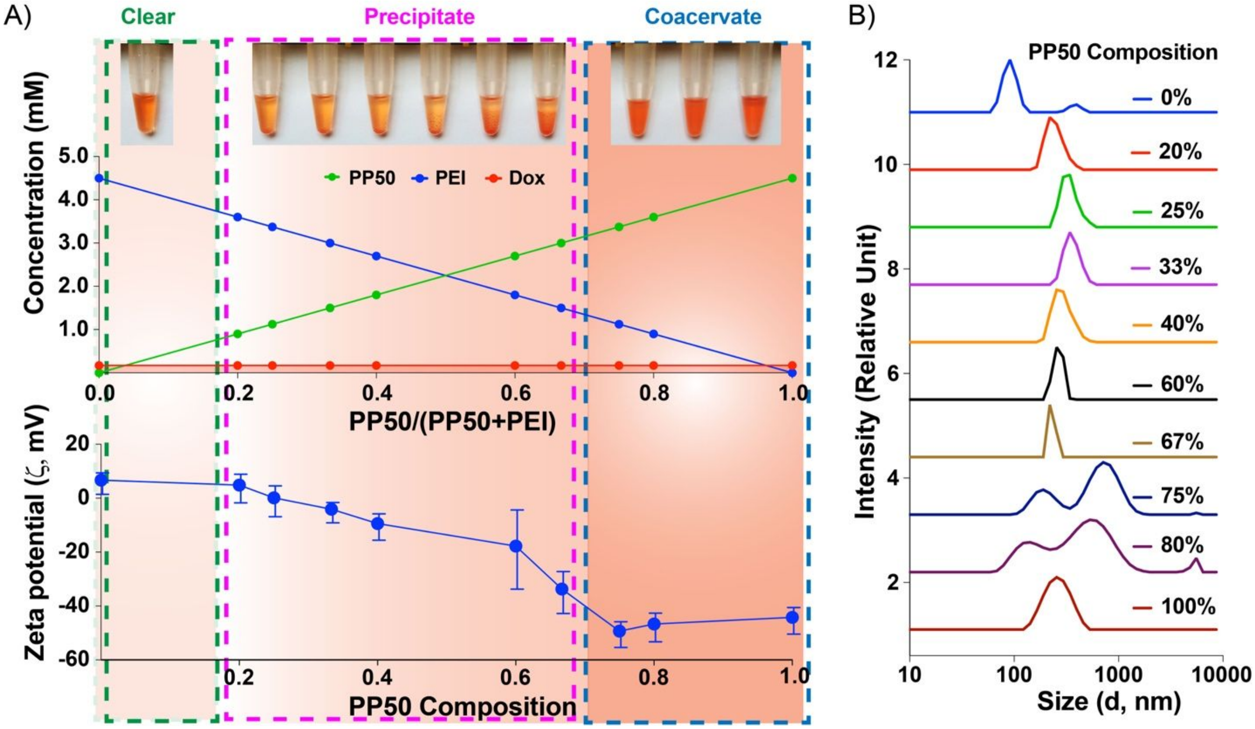

Figure S6. (A) Photographs of different formulas made with various PP50/PEI ratios are listed in the top panel. Clear solution was obtained with only PEI + Dox, precipitation was obtained with PP50 composition from 0.2 to 0.67, and turbid mixtures were obtained with PP50 composition greater than 0.75. Respective ζ value of complexes/coacervates-like system with different formulations are exhibited in the bottom panel. (B) Hydrodynamic diameters of complexes/coacervates-like system measured by DLS (PP50 composition is defined as [PP50] / ([PP50] + [PEI]))

Morphological characterization of coacervate-like system

Morphology of the Coacervate-like system

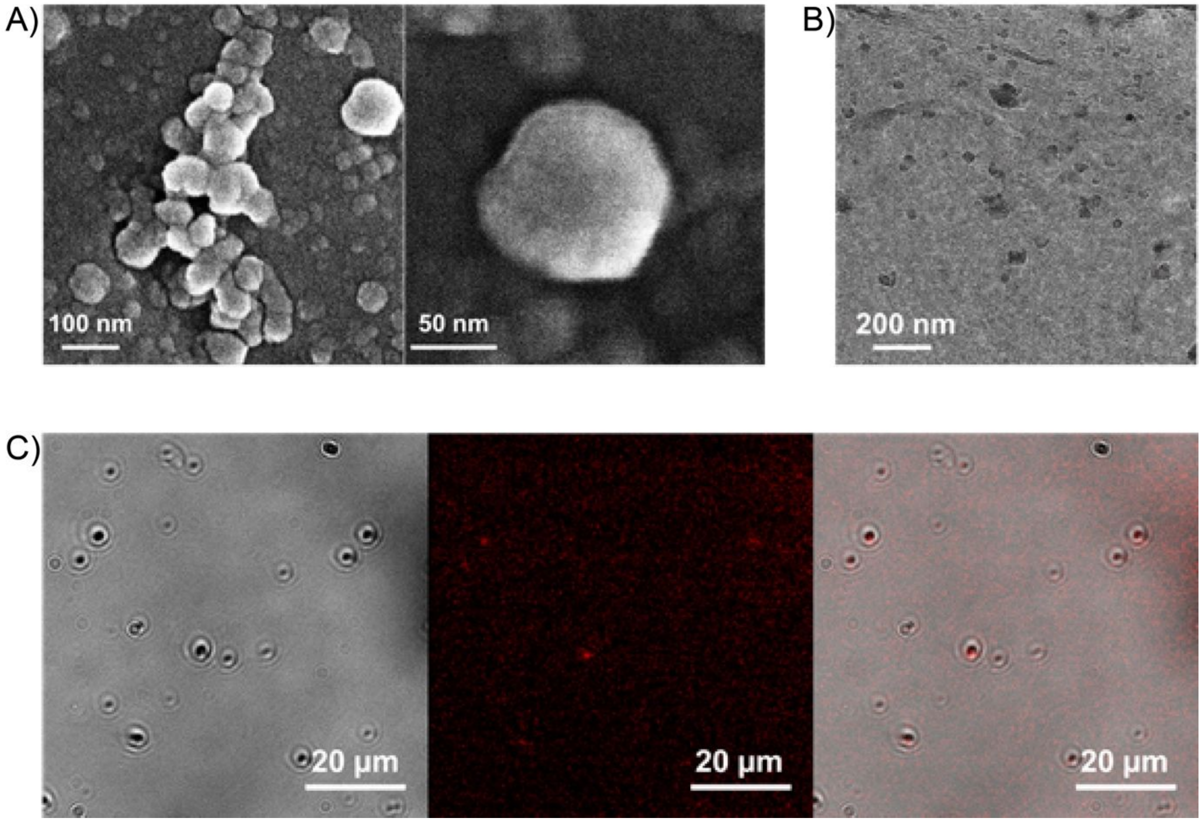

Figure S7. (a) SEM of coacervate-like system made with PP75 to PEI ratio at 4:1 and Dox concentration at 0.1mg/mL (b) TEM image of coacervate-like system made with PP50 to PEI ratio at 4 to 1 and Dox concentration at 0.1mg/mL (c) Bright field and fluorescence field (Dox) images of coacervate-like system made with PP50 to PEI ratio at 4 to 1 and Dox concentration at 0.1mg/mL.

Scattering signals detected by the nanoparticle tracking analyser – fluorescence filter

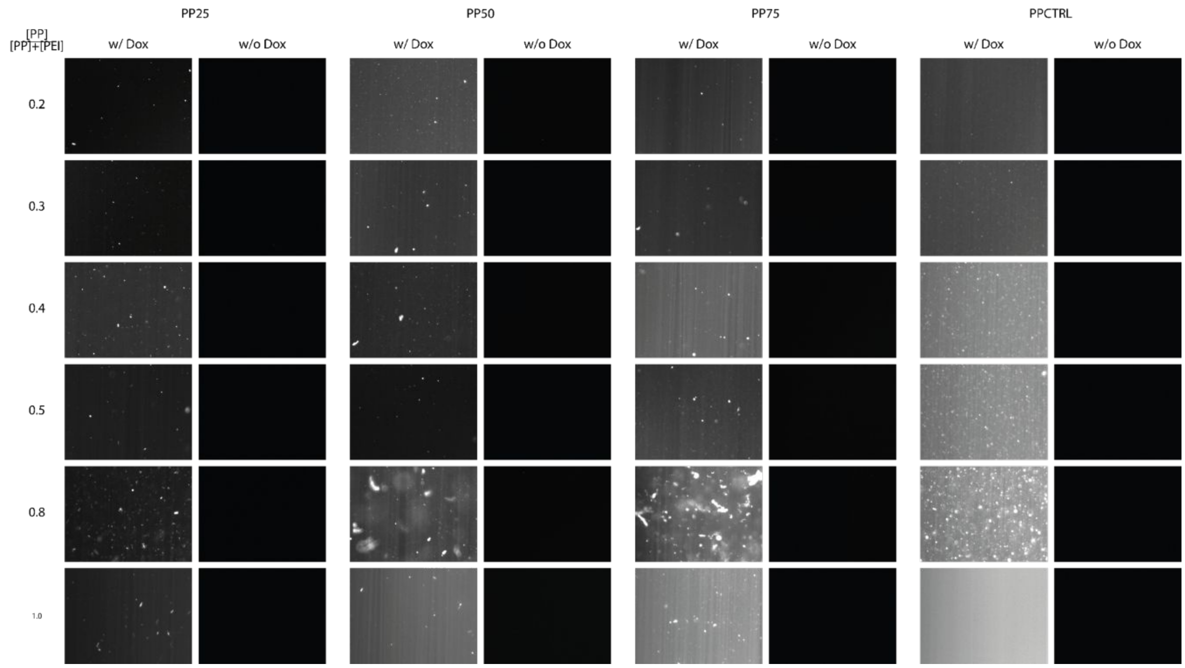

Figure S8. Fluorescence-filtered scattering signals recorded on a ZetaView™ Twin nanoparticle tracking analyser (NTA) equipped with a 540 nm wavelength fluorescence filter and a 520 nm excitation laser placed at a 90° angle. The complexes were injected into the analyser's quartz cell 2 minutes after preparation without further dilution and they were compared to equivalent complexes without doxorubicin

The signals for the mixes were compared with and without the presence of Dox. There was no signal detected for Dox-negative samples, but the mixes of PP polymers with Dox and PEI showed positive fluorescence signals indicating encapsulation of Dox within the resulting light-scattering complexes (i.e., ratios 0.2 – 0.8). It was also apparent that even without PEI, the Phe-modified PP polymers were able to interact and self-assemble with Dox and produce a fluorescence signal (i.e., ratio 1.0). This was not the case for the control PP polymer which was unable to form a complex with Dox without the presence of PEI.

Comparison of loading of Dox between PP50 and PP75

PP comparison for drug loading

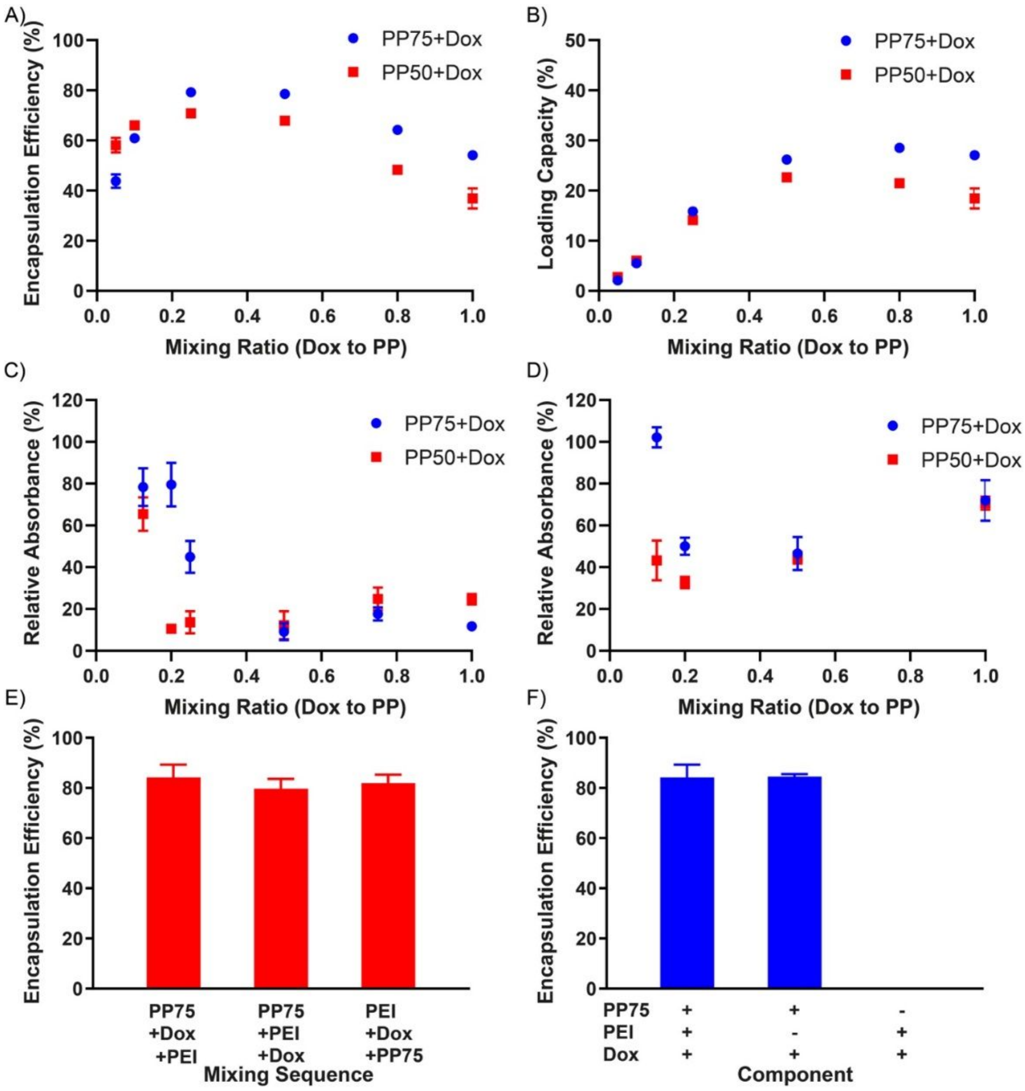

Figure S9. (a) Encapsulation efficiency and (b) loading capacity of coacervates-like system/complexes made with various ratios of PP75+Dox and PP50+Dox without PEI. Stability of coacervates-like system made with various ratios of PP75+Dox and PP50+Dox in (c) PBS and (d)10 mg/mL BSA. Stability was evaluated by recording changes in UV-VIS absorbance of the supernatant at 480 nm after allowing samples to stand for 24 hr. Encapsulation efficiency of Dox in PP75-PEI-Dox coacervate-like system fabricated with (e) various mixing ratio and (f) different mixing components (n = 3).

The difference between using PP75 and PP50 was compared with PP-Dox system. Dox encapsulation efficiency was measured with various Dox to PP polymer ratios (**Figure S9a**). At mixing ratios smaller than 0.75, PP75 can load Dox at a slightly higher rate than PP50. The encapsulation of PP75 decreases from a mixing ratio of 0.75 to 1. From the loading capacity data

(**Figure S9b**), it can be inferred that PP75 has reached full loading capacity after at mixing ratio of 0.8 and maximum encapsulation efficiency was achieved between 0.25 and 0.5 mixing ratio. PP50 also reached maximum loading capacity at 0.5 and the highest loading efficiency is accomplished with a similar mixing ratio as PP75. The stability of PP-Dox coacervates-like system was studied both in PBS solution and in presence of BSA. Upon mixing, homogeneous emulsions were obtained with all mixing ratios. However, some formulas underwent bulk phase separation overnight. The relative stability is evaluated by measuring the absorbance intensity at 480 nm right after the coacervates-like system is made and again after allowing samples to free stand for 24 hours. In PBS, PP75-Dox coacervate-like system shows better stability than PP50-Dox under 0.1, 0.2, and 0.25 mixing ratios. BSA was also added to the system to better mimic physiological condition, as protein adsorption can greatly affect the stability of nanoparticles. [2] The stability of all PP75-Dox and PP50-Dox coacervates-like system are improved with the addition of BSA. PP75-Dox again shows better stability than PP50-Dox system, and best stability is observed with PP75-Dox at 1 to 8 (Dox to PP75) ratio.

The mixing sequence of PP75-PEI-Dox coacervate-like system on encapsulation efficiency was also studied and summarized in **Figure S9f**. The highest encapsulation is reached when PP75 is first mixed with Dox then followed by the addition of PEI. By allowing PP75 and Dox to complex first and then adding PEI to the system, more Dox is brought into the system by PEI. However, the difference is very small, indicating the process is largely thermodynamically controlled. As shown in **Figure 2a**, coacervate-like system can be obtained without PEI. PP75-PEI-Dox, PP75-Dox, and PEI alone were compared for their loading efficiency (**Figure S9e & f**). The PP75-PEI is capable of encapsulating 84.2±5.0% of feeding Dox, slightly lower than the 84.5±1.0% efficiency achieved by PP75 alone. Dox release profile was established with PP75-PEI-Dox and PP75-Dox system under various pH values (**Figure S9f**). The result shows that releasing of Dox is slightly faster from PP75-PEI-Dox than from PP75-Dox.

Cytotoxic effects of Dox delivered by the coacervate-like structures system

Biocompatibility of individual components

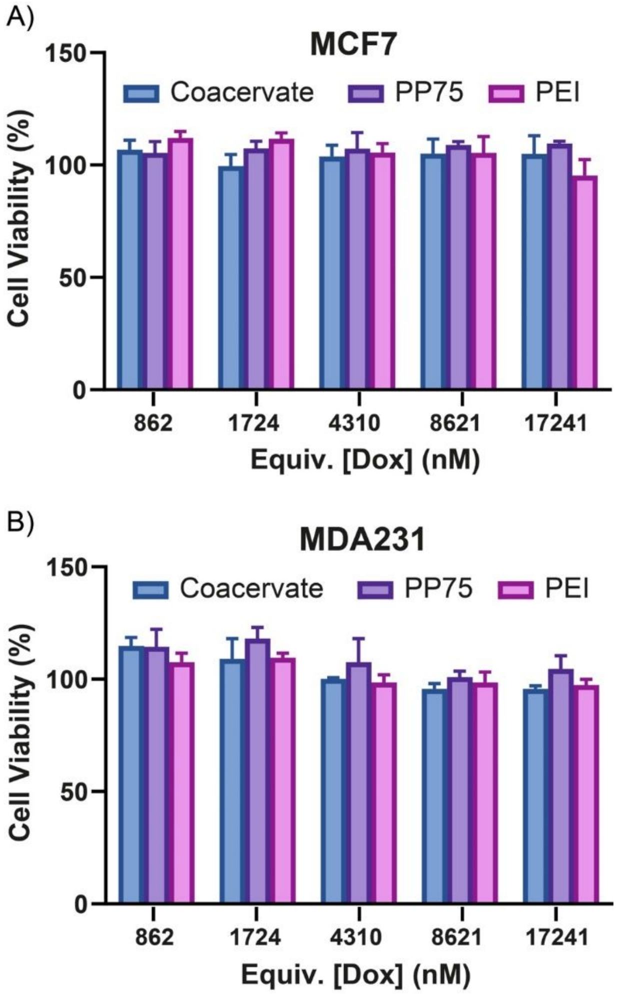

Figure S10. Cytotoxic effects of delivering agents PP75-PEI, PP75, and PEI measured with MTS assay against (A) MCF7 and (B) MDA231 (n = 3).

PP75-PEI-Dox, PP75-Dox, PEI-Dox, and free Dox were used to treat three distinct breast cancer cell lines, MCF7, MDA231, and T47D with 48- and 72-hour periods (**Figure S10**). First, MTS assay is performed with MCF7 and MDA231 cell lines treated with empty delivery agents to ensure there is no cytotoxicity effect of the carriers. In almost all cases, delivery agents improve the efficacy of Dox, except for MDA231 (72hr), where all four systems resulted in similar dose-response curves. The IC_{50} values are summarized in Table S11g. In MCF7, PP75-Dox and PEI-Dox have similar efficacies and both are even more effective than the PP75-PEI-Dox for both 48-

and 72-hour treatments. This means by combining the PP75 and PEI the delivery efficiency is somehow compromised for MCF7. In MDA231, PP75-Dox is more efficacious than PP75-PEI-Dox which is more efficacious than PEI-Dox for the 48-hour treatment. In T47D, PP75-PEI-Dox has the highest efficacy and PP75-Dox has similar performance but slightly higher IC_{50} values. The PEI-Dox despite having a smaller IC_{50} than free Dox, its dose-response curve is very similar to that of the free Dox, especially at the higher dose range. However, PEI-Dox is a simple mixture rather than a delivery system because PEI does not complex with Dox. To summarise, PP75-PEI and PP75 are both able to conjugate with Dox and improve its efficacy with three different breast cancer cell lines, even though the degree of improvement of the two systems differs in different cell lines.

Coacervate-like system response to 2D monolayer cells

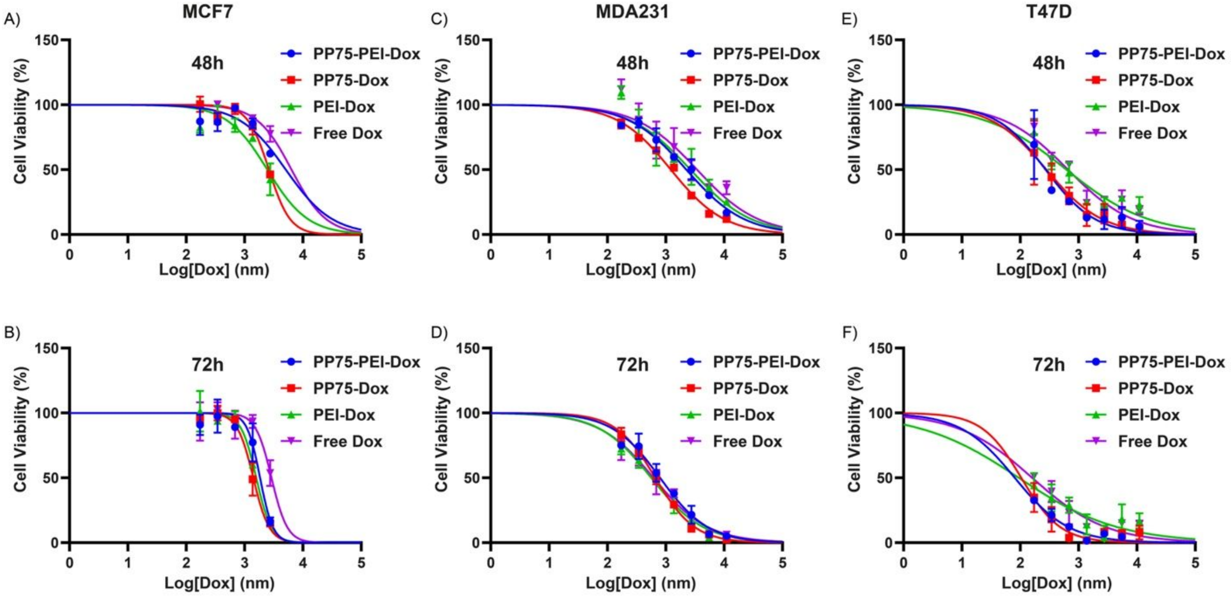

G)

Formulas	MCF7		MDA231		T47D	
	48 h	72 h	48 h	72 h	48 h	72 h
PP75-PEI-Dox IC_{50} (nm)	5108	1848	2254	798.2	272.8	80.76
PP75-PEI-Dox IC_{50} (nm)	2621	1415	1223	664.4	281.3	104.3
PP75-PEI-Dox IC_{50} (nm)	2464	1590	2650	565.3	619.7	94.63
PP75-PEI-Dox IC_{50} (nm)	6395	2901	3438	616.1	659.5	160.6

Figure S11. Dose-response curves measured by MTS assay with 2D monolayer of MCF7 (a) & (b), MDA231 (c) & (d), and T47D (e) & (f) breast cancer cell lines treated with PP75-PEI-Dox, PP75-Dox, PEI-Dox, and free, respectively Dox at 48 and 72 hours. The IC_{50} values are summarized in the tabular form (g) (n = 3).

Structured illumination and confocal microscopic studies

Structured illumination microscopy for sub-cellular localisation

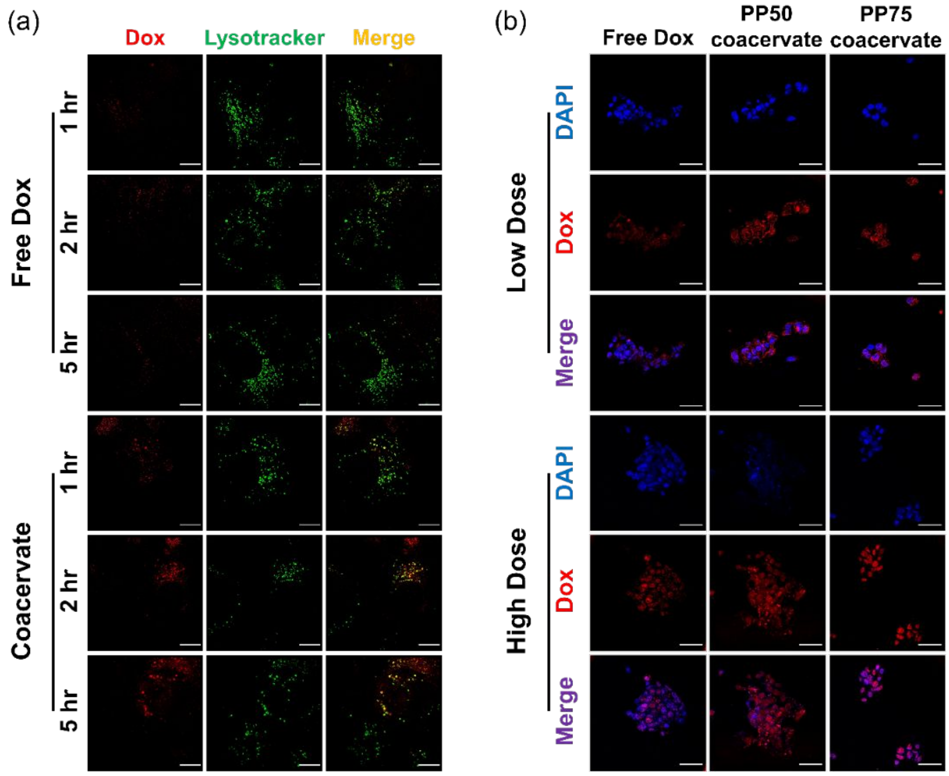

Figure S12. (a) Structured illumination microscopy (SIM) of MCF7 treated with either free doxorubicin or coacervate-like system (red fluorescence) and incubated with Lysotracker Green (green fluorescence) at 1, 2, and 5 hours, showing the subcellular distribution of free Dox and coacervate-like system. Scale bar represents 10µm. (b) Localization and uptake of coacervate-like system (fabricated at 80% [PP]/([PP]+[PEI])) delivered Dox by MCF breast cancer cells. Confocal microscopic imaging of MCF7 cells treated with coacervate-like system and free Dox at low (0.8 µg/mL) and high (1.6 µg/mL) doses after 12 hours. Scale bar represents 50 µm.

Coacervate-like delivery systems made with PP50 and PP75, both at [PP] / ([PP] + [PEI]) = 0.8, were subjected to MCF7 cell line to study their localisation using confocal fluorescence microscopy (**Figure S12**). The difference in localisation of Dox among free Dox, PP50 coacervate-like, and PP75 coacervate-like system was not super prominent. However, Dox fluorescence intensity was slightly higher with PP50 and PP75 coacervates-like system than with free dox at respective dosage. Higher dosage also resulted in higher fluorescence in the nuclei.

Investigation of delivery effects of the coacervate-like system in 3D multicellular spheroids

Functional validation of coacervate-like system with 3D spheroids

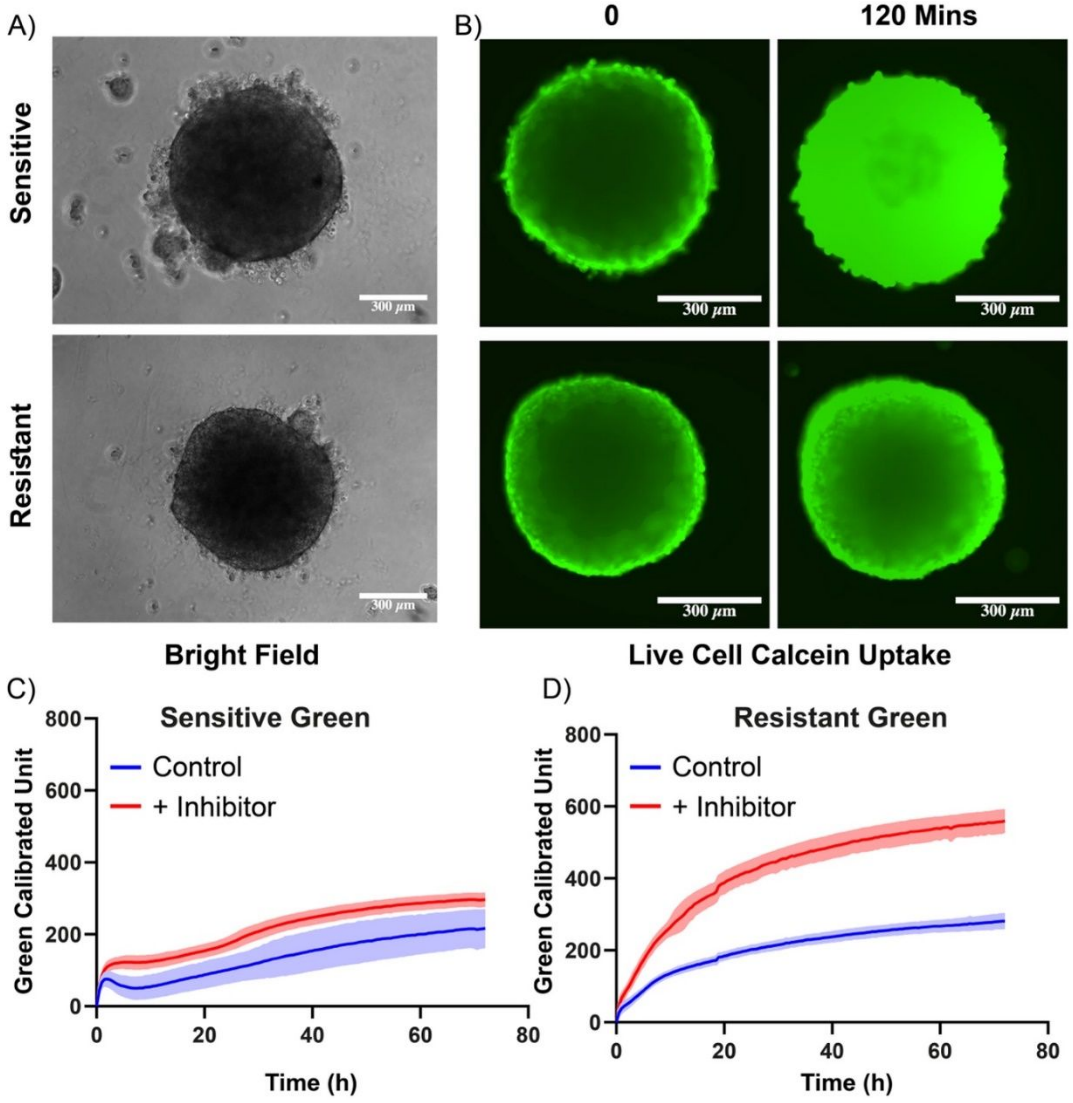

Figure S13. Validation of 3D tumor Spheroid from pre-treated patient's cancer cell. (a) The corresponding bright field microscopic images of spheroids. (b) Confocal images of 3D tumor spheroids made with drug-sensitive and drug-resistant cancer cells treated with calcein at 0 and 120 minutes. Raw total green fluorescence intensity in the field of view as acquired by the microscope of control groups (-/+ verapamil inhibitor) measured over 72 hours of calcein-AM treatment in (c) sensitive and (d) resistant spheroids during real-time imaging of 3D spheroids. Error bands represent standard deviations of 3 independent replicates (n = 3).

The sensitive and resistant spheroids were treated with a calcein-AM probe and confocal images were taken at 0-and 120minutes time points (**Figure S13b**). The resistant spheroid shows much

less calcein accumulation than the sensitive one at 120 minutes. The microscope acquired green fluorescence intensity (total fluorescence in the field of view) data was analysed during the real-time live cell imaging (**Figure S13c & d**). The control groups were treated only with calcein-AM with no drug treatment, and the "+inhibitor" groups were treated with both calcein and verapamil, which inhibits the activity of the efflux pump, such as P-glycoprotein, by blocking their calcium channels. The resistant spheroids showed a larger increase in green fluorescence with the addition of inhibitor, confirming the presence of MDR efflux pump in the cell line. Interestingly, the sensitive spheroids also demonstrated some elevation in green fluorescence intensity, also indicating the existence of MDR proteins. However, the difference in the degree of enhancement, verified that there was much higher level of overexpression of MDR proteins in the resistant (LK1108) cell lines than in the sensitive (LK0917) cell line.

Comparative analysis of coacervate-like system in 2D and 3D cancer spheroids

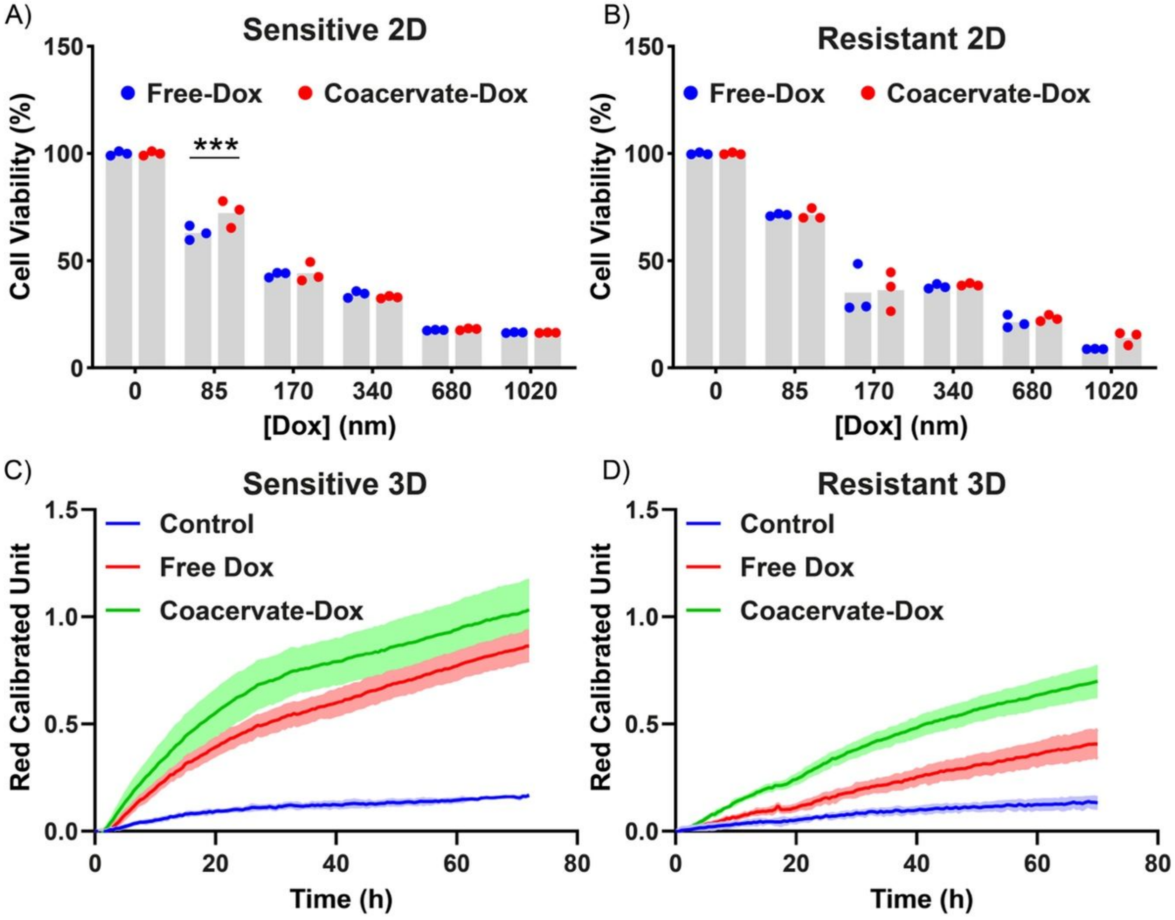

Figure S14. Cytotoxic effects of free Dox and coacervate-like-Dox system in (a) sensitive (LK0917) and (b) resistant (LK1108) cell lines in 2D monolayer (***$p \leq 0.001$). Raw total red fluorescence intensity in the field of view as acquired by the microscope in (c) sensitive and (d) resistant spheroids treated with free Dox and nanocoacervate-like Dox system over 72-hour treatment during real-time imaging of 3D spheroids. Error bands represent standard deviations of 3 independent replicates (n = 3).

The cytotoxic effect of free Dox and coacervate-like system delivered Dox was also investigated in 2D monolayer of sensitive (LK0917) and (b) resistant (LK1108) cells (**Figure S14a & b**). The coacervate-like system delivered Dox showed comparable cell viability in both drug-sensitive and drug-resistant cell lines as the free Dox. The coacervate-like system was even slightly less cytotoxic than the free Dox at 85 nM treating dose in sensitive cells. This result is similar to that

observed with MDA231 cells at 72-hour treating time. It is understandable that the delivery system did not result in enhanced cytotoxicity in drug-sensitive cell lines, as the free diffusion of Dox is sufficient to kill most of the sensitive cell. In terms of the resistant cells, the result suggests that 2D model is insufficient in fully reflecting the MDR nature of cell line. Therefore, the 3D model, which more closely resembles the complexity of a tumour, was selected as the principal model to study the delivery effects of coacervate-like system.

Realtime drug penetration

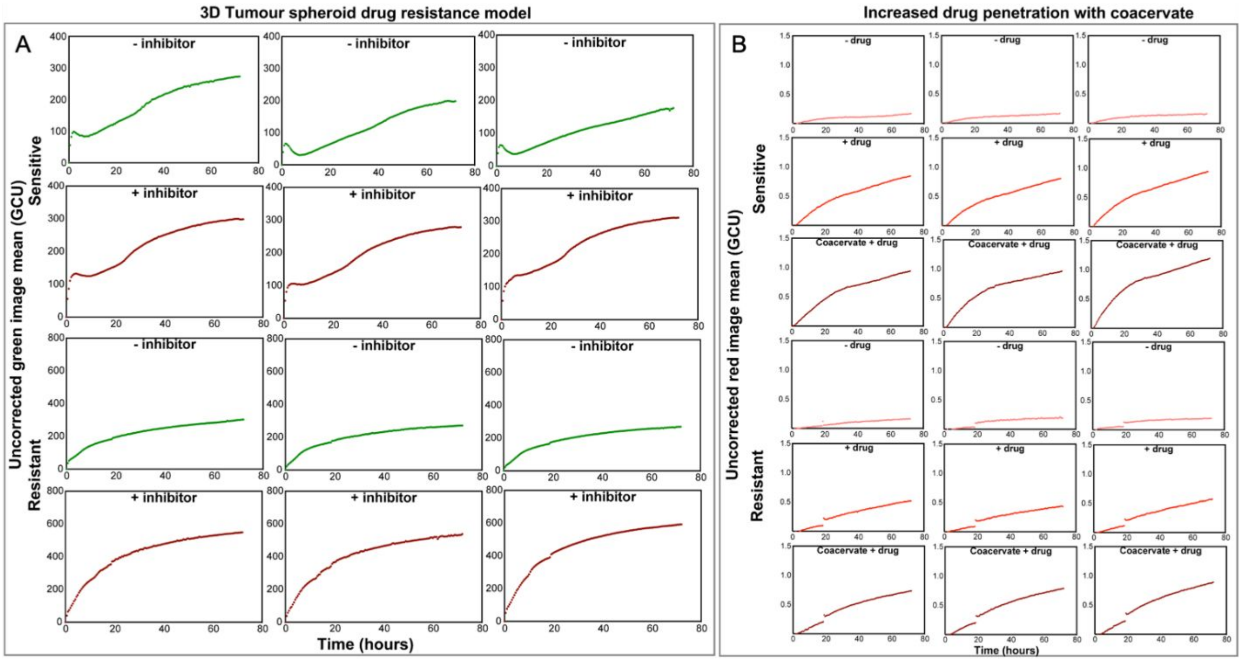

Figure S15. Drug penetration: (A) Sensitive and resistant tumour spheroids were treated with Calcein AM (+/-) Verapamil (MDR1 efflux pump inhibitor). Real-time live-cell fluorescence imaging of the spheroids was obtained over a period of 72 hours. Green fluorescence intensity corresponding to calcein uptake is shown here as obtained from the Incucyte Zoom. Each panel represents a spheroid. (B) Live-cell real -time imaging of the spheroids treated with the drug (+/-) coacervate-like structures is shown here for sensitive and resistant spheroids. The images were acquired over a treatment window of 72 hours, and images were taken at an interval of 30 minutes. The panel represents one individual spheroids.

The microscope acquired red fluorescence intensity (total fluorescence in the field of view) data was analysed during the real-time live cell imaging (**Figure S13c & d**). The sensitive spheroids

showed much higher Dox retention than the resistant spheroids as expected. In both cases, the nanocoacervate-like delivery system was able to improve the accumulation of Dox as displayed in red fluorescence intensity in the field of view (**Figure S14c &d and Figure S15B**). The degree of improvement was again much more noticeable in the resistant spheroids than the sensitive spheroids.

References:

(1) Munnier, E.; Cohen-Jonathan, S.; Linassier, C.; Douziech-Eyrolles, L.; Marchais, H.; Soucé, M.; Hervé, K.; Dubois, P.; Chourpa, I. Novel Method of Doxorubicin–SPION Reversible Association for Magnetic Drug Targeting. *Int. J. Pharm.* **2008**, *363* (1–2), 170–176. https://doi.org/10.1016/j.ijpharm.2008.07.006.

(2) Suvarna, M.; Dyawanapelly, S.; Kansara, B.; Dandekar, P.; Jain, R. Understanding the Stability of Nanoparticle–Protein Interactions: Effect of Particle Size on Adsorption, Conformation and Thermodynamic Properties of Serum Albumin Proteins. *ACS Appl. Nano Mater.* **2018**, *1* (10), 5524–5535. https://doi.org/10.1021/acsanm.8b01019.
